# Integrated models of blood protein and metabolite enhance the diagnostic accuracy for Non-Small Cell Lung Cancer

**DOI:** 10.1186/s40364-023-00497-2

**Published:** 2023-07-20

**Authors:** Runhao Xu, Jiongran Wang, Qingqing Zhu, Chen Zou, Zehao Wei, Hao Wang, Zian Ding, Minjie Meng, Huimin Wei, Shijin Xia, Dongqing Wei, Li Deng, Shulin Zhang

**Affiliations:** 1 Department of Immunology and Microbiology, Shanghai Jiao Tong University School of Medicine, Shanghai, 200025 China; 2 Department of Clinical Laboratory, Renji Hospital, Shanghai, 200001 China; 3 School of Life Science and Technology, Wuhan Polytechnic University, Wuhan, 430000 China; 4 Department of Clinical Laboratory, Children’s Hospital of Shanghai, Shanghai, 200040 China; 5 School of Biosciences and Biopharmaceutics, Guangdong Pharmaceutical University, Guangzhou, 510006 China; 6 Shanghai Cellsolution Biotech Co.,Ltd, Shanghai, 200444 China; 7 Department of Geriatrics, Huadong Hospital, Shanghai Institute of Geriatrics, Fudan University, Shanghai, 200040 China; 8 Department of Bioinformatics, School of Life Science and Biotechnology, Shanghai Jiao Tong University, Shanghai, 200240 China; 9 Zhongjing Research and Industrialization Institute of Chinese Medicine, Zhongguancun Scientific Park, Nanyang, 473006 Henan China; 10 Shanghai Public Health Clinical Center, Fudan University, Shanghai, 201508 China

**Keywords:** NSCLC, Blood, Proteomics, Metabolite, Integrated model

## Abstract

**Background:**

For early screening and diagnosis of non-small cell lung cancer (NSCLC), a robust model based on plasma proteomics and metabolomics is required for accurate and accessible non-invasive detection. Here we aim to combine TMT-LC-MS/MS and machine-learning algorithms to establish models with high specificity and sensitivity, and summarize a generalized model building scheme.

**Methods:**

TMT-LC-MS/MS was used to discover the differentially expressed proteins (DEPs) in the plasma of NSCLC patients. Plasma proteomics-guided metabolites were selected for clinical evaluation in 110 NSCLC patients who were going to receive therapies, 108 benign pulmonary diseases (BPD) patients, and 100 healthy controls (HC). The data were randomly split into training set and test set in a ratio of 80:20. Three supervised learning algorithms were applied to the training set for models fitting. The best performance models were evaluated with the test data set.

**Results:**

Differential plasma proteomics and metabolic pathways analyses revealed that the majority of DEPs in NSCLC were enriched in the pathways of complement and coagulation cascades, cholesterol and bile acids metabolism. Moreover, 10 DEPs, 14 amino acids, 15 bile acids, as well as 6 classic tumor biomarkers in blood were quantified using clinically validated assays. Finally, we obtained a high-performance screening model using logistic regression algorithm with AUC of 0.96, sensitivity of 92%, and specificity of 89%, and a diagnostic model with AUC of 0.871, sensitivity of 86%, and specificity of 78%. In the test set, the screening model achieved accuracy of 90%, sensitivity of 91%, and specificity of 90%, and the diagnostic model achieved accuracy of 82%, sensitivity of 77%, and specificity of 86%.

**Conclusions:**

Integrated analysis of DEPs, amino acid, and bile acid features based on plasma proteomics-guided metabolite profiling, together with classical tumor biomarkers, provided a much more accurate detection model for screening and differential diagnosis of NSCLC. In addition, this new mathematical modeling based on plasma proteomics-guided metabolite profiling will be used for evaluation of therapeutic efficacy and long-term recurrence prediction of NSCLC.

**Supplementary Information:**

The online version contains supplementary material available at 10.1186/s40364-023-00497-2.

## Background

Lung cancer is the global leading cause of cancer death [1]. Most patients were not diagnosed timely until stage IIIA or IV with 5-year survival rate 13% only. Since early diagnosis at stage I can increase the survival rate by 61% [2], simple and accurate screening and differential diagnostic approaches for lung cancer are critical [3]. Although advanced low-dose computed tomography (LDCT) has been used for early screening and differential diagnosis of lung cancer [4], its utility is still limited by the concern of radiation. Moreover, distinguishing between lung cancer and benign pulmonary diseases by imaging is so far a challenging problem [5]. Recently, plasma biomarkers of cancers have been highly sought-after features for cancer detection due to lower side-effects and cost [6]. Some potential blood biomarkers, including ctDNA [7] and microRNAs [8], have recently been evaluated for lung cancer diagnosis. While the genome and epigenome provide a blueprint for what may happen, however, the proteome provides certainty about what is going on because proteins and their modifications are the main final determinants for phenotype. Especially, metabolome as the downstream product of genome and proteome, allowing more accurate identification of disease-associated changes that have occurred, rather than predictions, can serve as direct biomarkers of biological processes and phenotypes [9, 10]. With the advancement of multi-omics technology and machine learning algorithms, it is now possible to discover more accurate features in blood for cancer detection. Recently, an integrative analysis of proteome, phosphoproteome, transcriptome, and whole-exome sequencing data revealed cancer-associated characteristics, such as tumor-associated protein variants and distinct proteomics features, and further study validated the plasma protein level of HSP 90β as a potential prognostic biomarker for lung adenocarcinoma [11]. By plasma metabolomic study of primary lung cancer, some low-molecular metabolites such as 1-salicylate glucuronide, adrenoyl ethanolamide, anabasine, dihydrocaffeic acid, 3-o-glucuronide, lysophosphatidyl glycerol, nicotinuric acid, o-arachidonoyl ethanolamine, and S-nitrosoglutathione were can be applied for the screening of lung cancer from healthy people. Compared with the most commonly used classic biomarkers, such as CEA, NSE, CYFRA21-1 and SCC, these newly reported potential biomarkers exhibited better sensitivity and specificity in their differential ability [12]. Although there are some limitations such as experimental sample size, no validation group set up to further judge the diagnostic efficiency, and no integration analysis of multi-omics of samples, these studies suggested a good application prospect of multi-omics integration analysis in the discovery of tumor markers.

In this study, we developed a new strategy to establish integrated models for enhancing the detection performance of NSCLC. Firstly, advanced TMT-LC-MS/MS was introduced to help find more DEPs in the plasma of NSCLC patients. Then Plasma proteomics-guided metabolites were selected for clinical evaluation. To improve the accuracy and robustness of the detection models, all the candidate biomarkers and classic tumor markers were quantified by quality-controlled commercial kits. Finally, high-performance integrative detection models for NSCLC were established using logistic regression algorithm. This integrated model consisting of blood proteins and metabolites significantly improved the diagnostic accuracy for NSCLC compared with single omics model and classic tumor markers. In addition, the integrated models provide supportive evidences for evaluation of therapeutic efficacy and recurrence prediction of NSCLC.

## Methods

### Study designs

**Fig. 1:**
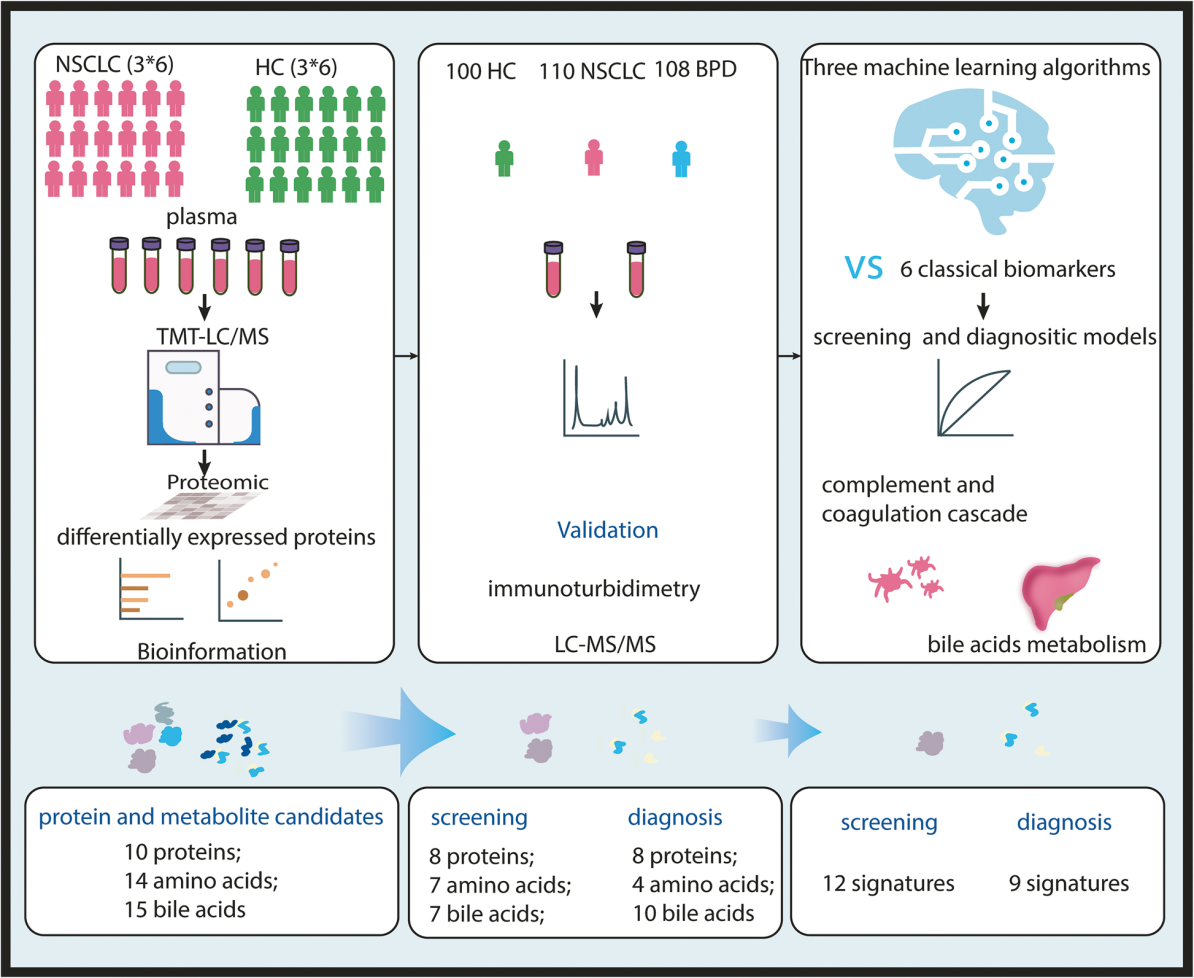
Overview of screening and diagnostic model development for NSCLC

To identify DEPs, we performed tandem mass tag labeling and liquid chromatography/mass spectrometry (TMT-LC-MS/MS) on three-paired plasma samples (NSCLC vs. HC, Fig. 1). Each sample was a mixture of six individuals (3 men and 3 women). The ages are approximately matched (NSCLC, 36 ~ 78 years old, and HC, 30 ~ 75 years old). According to the analysis of Gene Ontology (GO) terms and Kyoto Encyclopedia of Genes and Genomes (KEGG) pathways for DEPs in plasma, we selected potential protein and metabolite markers for clinical evaluation. The details of validated assays are accessible in appendix (Supplementary Table 1). Three supervised machine learning algorithms fitted the validated features to establish the high-performance model for lung cancer detection.

### Demographics of study cohorts

A total of 318 patients and controls were enrolled, including 110 NSCLC, 100 HC, and 108 BPD in Shanghai Jiao Tong University School of Medicine Affiliated Renji Hospital from October 2020 to January 2021. All plasma/serum samples werestored at -80℃. All NSCLC patients were diagnosed with primary NSCLC by imaging and histopathological examinations with no sign of digestive tract metastasis. In HC group, CT or X-ray examination showed no apparent lesions in the lungs, no noticeable lesions in other organs, and abnormalities in common indicators such as blood sugar, blood lipids, and liver and kidney function. The BPD group was clinically confirmed, and the possibility of lung tumors and gastrointestinal diseases was ruled out. All of the enrolled patients didn’t take immunosuppressants and amino acid drugs. There was no statistically significant difference in age and gender between the groups (*P >* 0.05) (Table 1).

**Table 1:** Clinical characteristics of patients

	NSCLC Group	HC Group	BPD Group
**Total**	110	100	108
**Sex**			
Male	68	60	57
Female	42	40	51
**Age, y**			
Mean (SD)	61.4 (12.2)	59.5(14.2)	64.1(8.6)
Range	27–82	24–81	25–89
**TNM Stage**			**Type of BPD**
I	56(50.9%)	─	Tuberculosis 30 (27.8%)
II	11(10.0%)	─	Lung nodules 18 (16.7%)
III	17(15.5%)	─	Non-TB lung infection 40 (37.0%)
IV	24(21.8%)	─	Others 20 (18.5%)
Unknown	2(1.8%)	─	
**Histological type**			
LUAD	88(80.0%)	─	
LUSC	20(18.2%)	─	
Other NSCLC	2 (1.8%)	─	

*Abbreviations*: *SD *Standard deviation, *NSCLC *Non-small cell lung cancer, *HC *Health control, *BPD *Benign pulmonary disease, *LUAD *Lung adenocarcinoma, *LUSC *Lung squamous cell carcinoma

The plasma samples were collected using EDTA anticoagulation tubes, and serum samples were collected using coagulation tubes. This study was approved by the Ethics Committee of Shanghai Jiao Tong University and conformed to the Declaration of Helsinki. All patients provided informed consent before they were included in this study.

### Statistical analysis

SPSS 22.0 software and MedCalc 15.0 software was used for statistical analysis. GraphPad Prism 8.4.2 was used for scatter plot display. K-S test was used for normal distribution test. Normally distributed data is represented by x ± s, and independent-sample t-test is used for comparison between groups; measurement data that is non-normally distributed is represented by M (Q1, Q3), and Mann-Whitney U test is used for comparison between groups.

### Bioinformatics analysis

Proteomics data from mixed plasma samples was calculated by the two-sample two-tailed T test. DEPs were identified with fold change > 1.20 or < 0.833 and *P* value ≤ 0.05. We performed the Gene Ontology (GO) enrichment analysis using Metascape (https://metascape.org/) for gene annotation and analysis, and we got the result of biological process (BP), cellular component (CC), molecular function (MF) and Kyoto Encyclopedia of Genes and Genomes (KEGG) pathway. The *P* value < 0.05 was regarded as significant. The STRING (Search Tools for the Retrieval of Interacting Genes (http://www.string-db.org/) was employed to calculate protein-protein interaction network (PPI), and then PPI created file was visualized using Cytoscape (version 3.7.2, http://www.cytoscape.org/).

### Clinical evaluation of candidate features

#### Plasma protein profiling

Ten proteins candidates, including ApoA1, ApoA2, ApoB, ApoC3, C1INH, C3, C4, Fg, FN, Lp(a), were validated by turbidimetric inhibition immune assay through the H7600 biochemical analyzer. The level of C1INH, C3, C4 were measured by turbidimetric inhibition immune assay through BN™ II specific protein analyzer. The CS-5100 hemostasis analyzer was used to detect the concentration of Fg by coagulation turbidimetric method.

#### Amino acid profiling

Add 10 µl serum in Eppendorf (EP) tubes, add 40 µl amino acid sample diluent, shake and mix at 2000 rpm for 5 min, then using nitrogen blowing instrument dry the sample at 50℃. Add 100 µl reconstituted solution to 96-well plate, shake and mix at 600 rpm for 5 min, use LC-20 A liquid chromatograph and API3200MD triple quadrupole mass spectrometer for detection, use Analyst mass spectrometer workstation collect data and mass spectrum images.

Chromatographic conditions: analytical column is ACE Excel3 C18 (3.0 mm×100 mm); column temperature is 40℃; mobile phase A is a mixture of ultrapure water and mobile phase additives; mobile phase B is a mixture of methanol and mobile phase additives; gradient elution; the flow rate is 550µL/min.

Mass spectrometry conditions: electrospray ion source, positive ion scanning, ion source parameters are atomizing gas pressure of 50 psi, auxiliary heater pressure of 50 psi, curtain gas pressure of 30 psi, collision gas pressure of 6 psi; ion source voltage of 5000 V; The ion source temperature is 500℃ for MRM scan analysis.

#### Bile acid profiling

Mix 100 µl serum sample and 500 µl extract containing internal standard, vortex and mix at 2500 rpm for 5 min, then centrifuge at 13,000 rpm, 10 min. Transfer 400 µl supernatant to a 96-well plate, using nitrogen blowing instrument dry the sample at 60 °C; add 100 µl of the reconstituted solution, place the 96-well plate in a microplate constant temperature shaker and mix at 700 rpm for 10 min, transfer the reconstituted solution in the 96-well plate to a special filter plate, and place a new one under the filter plate. Place the filter plate and 96-well plate together in a multi-tube rack automatic balance centrifuge to filter, centrifuge at 4000 rpm for 1 min, collect the filtrate, use LC-20 A liquid chromatograph and API3200MD triple quadrupole. The mass spectrometer is used for detection, and the Analyst mass spectrometry workstation is used to collect data and mass spectrometry images.

Chromatographic conditions: analytical column is XbridgeC18 (3.0 mm×50 mm); column temperature is 40℃; mobile phase A is a mixture of ultrapure water and mobile phase additives; mobile phase B is methanol; flow rate is 500µL/min. Mass spectrometry conditions: electrospray ion source, negative ion scanning, ion source parameters are atomizing gas pressure of 60psi, auxiliary heater pressure of 65 psi, curtain gas pressure of 20 psi, collision gas pressure of 8 psi; ion source voltage of -4500 V; The ion source temperature is 600 °C for MRM scan analysis.

### Detection of six classic lung cancer biomarkers

The Cobase801 electrochemiluminescence analyzer was used to detect six classic lung cancer markers (CA125, CA199, CEA, CYFRA211, NSE, SCC) using electrochemiluminescence immunoassay according to the clinical standard operation procedure.

### Proteins and amino acids related to NSCLC stage

To explore the relationship between candidates and NSCLC stages, NSCLC patients were divided into 67 cases in the early stage (stage I to II) and 41 cases in the middle and late stage (stage III to IV). The clinical staging was performed according to the eighth edition of tumor, node and metastasis classification [13]. Plasma ApoA1 and ApoA2 levels gradually decreased with the progression of the disease (*P* < 0.05); C1INH, C3, C4, and Fg gradually increased with the progression of the disease (*P <* 0.05) (Supplementary Fig. 6A. There was no significant difference between the remaining proteins in the early and middle and late stages of NSCLC. Serum levels of Ala, Glu, Lys, Pro, Val, and Cit gradually decreased with the progression of the disease (*P <* 0.05) (Supplementary Fig. 6B). The remaining small molecule metabolites had no significant difference in the early, middle and late stages of NSCLC.

### Machine learning algorithms modeling

#### Screening model establishment

Sample selection: 80% of the sample size was randomly portioned to form training samples, including 88 cases in the NSCLC group and 80 cases in the HC group. Twenty-five indicators with differences between the NSCLC group and the HC group were included in each analysis model.

1. The establishment of binary logistic regression model: the stepwise binary logistic regression analysis results show a 12 signatures panel which consist of ApoA2, ApoB, C3, FN, His, Cit, Orn, CA, UDCA, LCA, GCDCA, CEA are the main influencing factors for screening NSCLC. Based on this, the screening model${Y}_{1}=1/\left(1+{e}^{-logitP}\right)$ 
  where *logitP*=- 0.282*ApoA2* + 4.317*ApoB* + 3.948*C3–0*.006*FN* − 0.088*His* + 0.084*Cit* + 0.026*Orn* + 0.001*CA* − 0.071*LCA* − 0.004*UDCA* + 0.001*GCDCA* + 0.510*CEA* + 2.475, the model coefficient comprehensive test and Hosmer-Lemeshow test results show that the model fits well (*χ*^*2*^ = 149.37, *P* < 0.05; *χ*^*2*^ = 4.95, *P* > 0.05).
2. The establishment of Fisher discriminant analysis model: The results of stepwise Fisher discriminant analysis show that ApoA2, ApoB, His, Lys, Tyr, Val, Orn, LCA, GCDCA are the main influencing factors for screening NSCLC. Based on this, the screening model${Y}_{2}=1/\left(1+{e}^{-FisherP}\right)$, where *FisherP* = − 0.102*ApoA2* + 1.282*ApoB* − 0.036*His* − 0.011*Lys* − 0.022*Tyr* + 0.017*Val* + 0 .013*Orn* − 0.009*LCA* + 0.001*GCDCA* + 2.451. The Wilks-LAMBDA test results showed significant differences between the groups and good model fit (*χ*^*2*^ = 139.77, *P*<0.05).
3. The establishment of Bayes discriminant analysis model: The results of Bayes discriminant analysis by stepwise method show that ApoA2, ApoB, His, Lys, Tyr, Val, Orn, LCA, GCDCA are the main influencing factors for screening NSCLC. Based on this, the screening model ${Y}_{3}=1/\left(1+{e}^{-Bayes2NSCLC-Bayes3HC}\right)$, where *Bayes3HC* = 1.950*ApoA2* + 19.865*ApoB* + 0.192*His* + 0.035*Lys* + 0.242*Tyr* − 0.011*Val* − 0.009*Orn* − 0.013*LCA* + 0.001*GCDCA* − 57.274; *Bayes3NSCLC*=1.713*ApoA2* + 22.859*ApoB* + 0.106*His* + 0.009*Lys* + 0.190*Tyr* + 0.029*Val* + 0.021*Orn* − 0.009*LCA* + 0.001*GCDCA* − 51.422.
  Model performance analysis: Model${Y}_{1}$, ${Y}_{2}$, and ${Y}_{3}$ screen NSCLC with AUC of 0.959, 0.944, 0.944, respectively.
4. The establishment of single-omics or combined targeting multi-omics screening models: Here we mentioned other screening models we used to prove that multi-omics models are superior to single-omics model. All of these models were established by stepwise binary logistic regression. The screening model of metabolites:${Y}_{\alpha 1}=1/\left(1+{e}^{-logitP}\right)$, *logitP* = 0.008*Gly* − 0.093*His* + 0.033*Orn* − 0.026*LCA* + 0.001*GCDCA* + 1.121.

The screening model of metabolites + proteins:${Y}_{\beta 1}=1/\left(1+{e}^{-logitP}\right)$, *logitP* = − 0.304*ApoA2* + 3.156*ApoB* + 2.615*C3* + 0.011*Gly* − 0.084*His* + 0.028*Orn* − 0.003*UDCA* − 0.036*LCA* + 0.001*GCDCA* + 1.911.

The screening model of metabolites + classic markers:${Y}_{\gamma 1}=1/\left(1+{e}^{-logitP}\right)$, *logitP* = 0.056*Cit* − 0.078*His* + 0.028*Orn* − 0.051*LCA* + 0.001*GCDCA* + 0.455*CEA* + 0.354*CYFRA21-1–0*.336.

The screening model of proteins: ${Y}_{\delta 1}=1/\left(1+{e}^{-logitP}\right)$, *logitP* = − 0.252*ApoA2* + 2.598*ApoB* + 1.757*C3–0*.002*FN* + 0.667*FG* + 1.047.

#### Diagnostic model establishment

Sample selection: 80% of the sample size were randomly portioned to form a training sample, including 88 cases in the NSCLC group and 86 cases in the BPD group. Twenty-six indicators with differences between the two groups were included in each analysis model.

1. The establishment of binary logistic regression model: The stepwise method of binary logistic regression analysis results showed a 9 signatures panel which consist of ApoA2, Lp(a), C3, Fg, Cit, GDCA, TCDCA, CYFRA21-1, NSE are the main influencing factors in identifying NSCLC. Based on this, the identification model ${Y}_{4}=1/\left(1+{e}^{-logitP}\right)$, where *LogitP* = 0.141*ApoA2* + 0.003*Lp(a)* + 1.949*C3–0*.356*Fg* + 0.058*Cit* + 0.002*GDCA* − 0.002*TCDCA* + 0.206*CYFRA21-1* + 0.076*NSE* − 8.266, the model coefficient comprehensive test and Hosmer-Lemeshow test results show that the model fits well (*χ*^*2*^ = 135.04, *P*<0.05; *χ*^*2*^ = 7.60, *P*>0.05).
2. The establishment of Fisher discriminant analysis model: The results of stepwise Fisher discriminant analysis show that ApoA2, Lp(a), Cit, GDCA, TCDCA, CEA, CYFRA21-1 are the main influencing factors in identifying NSCLC, the identification model${Y}_{5}=1/\left(1+{e}^{-FisherP}\right)$, Where *FisherP* = 0.116*ApoA2* + 0.001*Lp(a)* + 0.042*Cit* + 0.001*GDCA* − 0.001*TCDCA* + 0.010*CEA* + 0.033*CYFRA21-1–4*.271. The Wilks-LAMBDA test results showed significant differences between the groups and good model fit (*χ*^*2*^ = 71.91, *P*<0.05).
3. The establishment of Bayes discriminant analysis model: The results of Bayes discriminant analysis including the stepwise method showed that ApoA2, Lp(a), Cit, GDCA, TCDCA, CEA, CYFRA21-1 were the main influencing factors in identifying NSCLC. Based on this, the identification model ${Y}_{6}=1/\left(1+{e}^{-Bayes6NSCLC-Bayes6BPD}\right)$, where *Bayes6NSCLC*=0.972*ApoA2* + 0.007*Lp(a)* + 0.240*Cit* + 0.001*GDCA* + 0.001*TCDCA* + 0.033*CEA*+ 0.138*CYFRA21-1–18*.203; *Bayes6BPD=*0.804*ApoA2* + 0.005*Lp(a)* + 0.179*Cit* + 0.001*GDCA* + 0.003*TCDCA* + 0.018*CEA* + 0.091*CYFRA21-1–11*.990.
  Model performance analysis: Model ${Y}_{4}$, ${Y}_{5}$, ${Y}_{6}$ identify NSCLC with AUC of 0.872, 0.859, 0.855 respectively.
4. The establishment of single-omics or combined targeting multi-omics diagnostic models: We mentioned other diagnostic models we used to prove that multi-omics models are superior to single-omics model. All of these models were established by stepwise binary logistic regression. The diagnostic model of metabolites:${Y}_{\alpha 2}=1/\left(1+{e}^{-logitP}\right)$, *logitP* = 0.006*Val* + 0.061*Cit* + 0.002*GDCA* − 0.003*TCDCA* − 3.732.

The diagnostic model of metabolites + proteins:${Y}_{\beta 2}=1/\left(1+{e}^{-logitP}\right)$, *logitP* = 0.064*Cit* + 0.002*GDCA* − 0.002*TCDCA* + 0.148*ApoA2* + 1.738*C3* + 0.002*Lp(a)* − 7.794.

The diagnostic model of metabolites + classic markers:${Y}_{\gamma 2}=1/\left(1+{e}^{-logitP}\right)$, *logitP* = 0.006*Val* + 0.053*Cit* + 0.002*GDCA* − 0.003*TCDCA* + 0.108*NSE* − 4.709.

The diagnostic model of proteins: ${Y}_{\delta 2}=1/\left(1+{e}^{-logitP}\right)$, *logitP* = 0.186*ApoA2* + 1.195*C3* + 0.002*Lp(a)* − 5.699.

## Results

### Quantitative proteomics analysis identified protein and metabolite features of plasma for modeling NSCLC detection

**Fig. 2:**
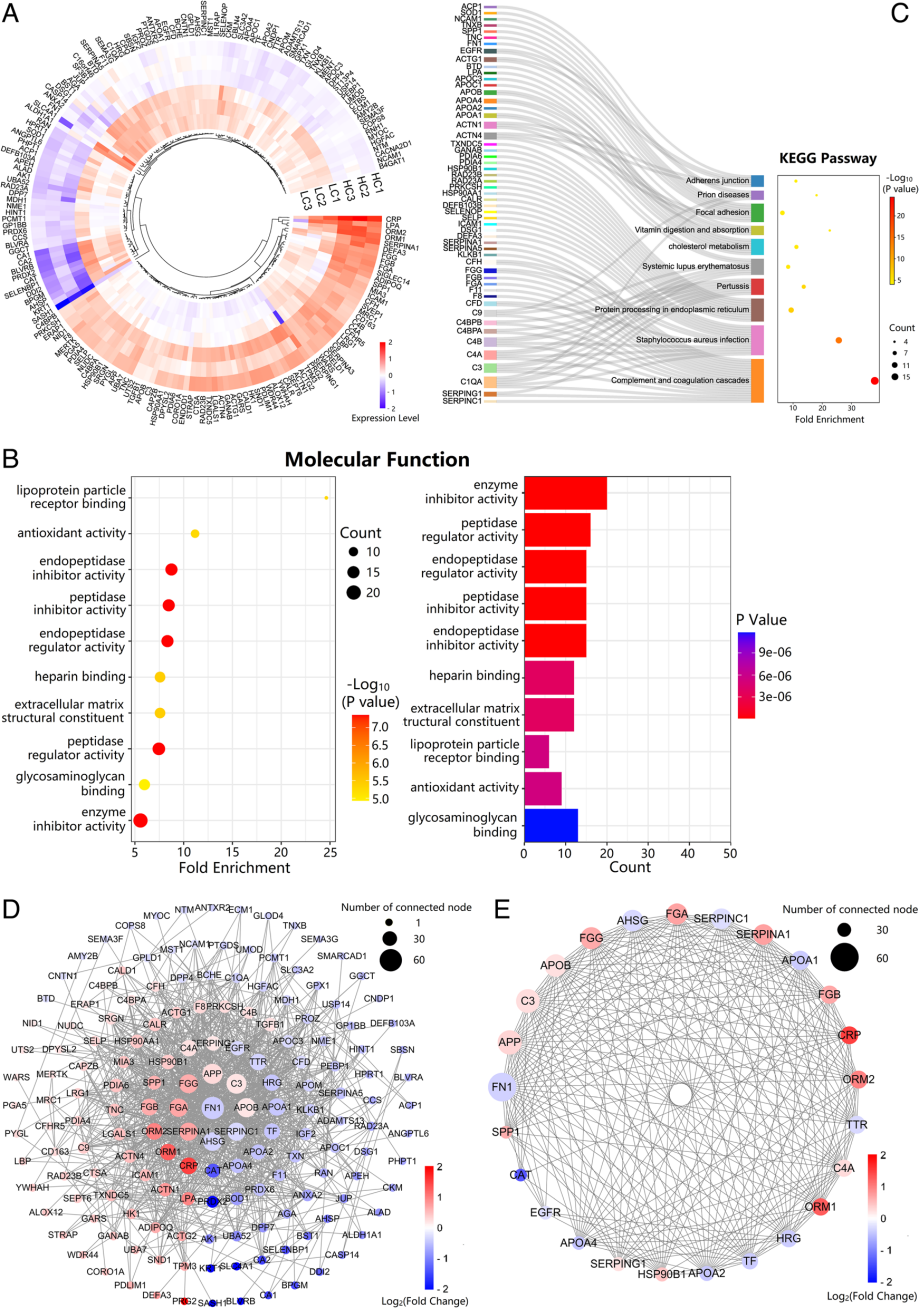
Differentially expressed proteins screening based on bioinformatics.** A** Heatmap of 180 DEPs with fold change > 1.2 or < 0.833 and *P* value ≤ 0.05 based on TMT-LC-MS/MS. **B** The molecular function of top fold enrichment is lipoprotein particle receptor binding. **C** Kyoto Encyclopedia of Gene and Genomes (KEGG) pathways of DEPs, which shows most DEPs are enriched in complement and coagulation cascades. **D** Protein-protein interaction (PPI) network. **E** The 25 hub proteins in PPI network

A total of 942 proteins were identified that could be quantified in both NSCLC and HC samples. There were 180 DEPs, including 77 up-regulated proteins with fold change greater than 1.2 and 103 down-regulated proteins with fold change less than 0.833 (Fig. 2A). GO analyses found that the molecular function of top fold enrichment is lipoprotein particle receptor binding (Fig. 2B). KEGG pathway analyses found that the top fold enrichment of pathways are the complement and coagulation, and cholesterol metabolism (Fig. 2C). Protein-protein interaction network analysis of 180 DEPs found 25 hub genes with the largest number of interactions among all sorted genes (Fig. 2D and E). Based on GO and KEGG analyses, we revealed the frequency of the top 10 proteins associated with biological process, cell components, molecule functions, and KEGG pathway, and then identified top 13 candidate biomarkers among the 25 candidate proteins (Supplementary Table 1). All of the top 13 candidate biomarkers are associated with tumorigenesis, including apolipoprotein A1 (ApoA1), apolipoprotein A2 (ApoA2), apolipoprotein B (ApoB), apolipoprotein C3 (ApoC3), fibrinogen alpha chain (FGA), fibrinogen beta chain (FGB), fibrinogen gamma chain (FGG), apolipoprotein (a) [Apo(a)], C1 inhibitor (C1INH), complement C3 (C3), complement C4A (C4A), complement C4B (C4B) and fibronectin (FN). The other 12 proteins among the 25 candidate proteins with low connection to top pathways are excluded. Based on the availability of quality-controlled commercial kits, we then confirmed 10 DEPs, involving complement and coagulation and lipoprotein particle binding proteins, including ApoA1, ApoA2, ApoB, ApoC3, C1INH, C3, C4, fibrinogen (Fg), FN, Lp(a) (Supplementary Table 1) for clinical evaluation. It was known that cholesterol is the substrate of bile acids. Also, amino acid metabolism in malignancy is aberrant [14], and plasma-free amino acid profile is different in cancer [15]. Based on plasma proteomics-guided metabolite profiling, we finally decided to choose bile acids as well as associated amino acids as clinical quantification targets, including 14 amino acids [Alanine (Ala), Arginine (Arg), Glutamic acid (Glu), Glycine (Gly), Histidine (His), Leucine (Leu), Lysine (Lys), Methionine (Met), Phenylalanine (Phe), Proline (Pro), Tyrosine (Tyr), Valine (Val), Citrulline (Cit) and Ornithine (Orn)], and 15 bile acids [Cholic acid (CA), Chenodeoxycholic acid (CDCA), Deoxycholic acid (DCA), Lithocholic acid (LCA), Ursodeoxycholic acid (UDCA), Glycocholic acid (GCA), Glycochenodeoxycholic acid (GCDCA), Glycodeoxycholic acid (GDCA), Glycolithocholic acid (GLCA), Glycoursodeoxycholic acid (GUDCA), Taurocholate acid (TCA), Taurochenodeoxycholic acid (TCDCA), Tauroursodeoxycholic acid (TDCA), Taurolithocholic acid (TLCA) and Tauroursodeoxycholic acid (TUDCA)]. In addition, 6 clinical classic tumor biomarkers, including CA12-5, CA19-9, CEA, CYFRA21-1, NSE, and squamous cell carcinoma antigen (SCC), were selected to be evaluated and integrated for establishing screening and diagnostic models.

### Feature candidates of proteins and metabolites were validated by clinical evaluation

Feature candidates consisting of 10 DEPs, 14 amino acids, 15 bile acids, and 6 classic tumor biomarkers were clinically evaluated by blood samples from 100 HC, 110 NSCLC, and 108 BPD (Table 2). Compared NSCLC with HC group, the plasma level of ApoA1, ApoA2, and FN in the NSCLC group decreased (*P <* 0.05), while the level of ApoB, C1INH, C3, C4, and Fg was increased (*P <* 0.05) (Supplementary Fig. 2). The plasma level of His, Lys, and Tyr in the NSCLC group was decreased (*P <* 0.05), and the Gly, Val, Cit, and Orn increased (*P <* 0.05) (Supplementary Fig. 3). The DCA, LCA, UDCA, GLCA, and TDCA in the NSCLC group were significantly reduced (*P <* 0.05), and the plasma levels of CA and GCDCA were increased (*P <* 0.05) (Table 2, Supplementary Fig. 4). The CA19-9, CEA, and CYFRA21-1 in the NSCLC group were significantly higher than in the HC group (*P <* 0.05) (Supplementary Fig. 5). In addition, other candidate features do not show significant differences (*P >* 0.05).

**Table 2:** The differentially expression of 14 serum amino acids, 15 bile acids, 6 classic tumor markers, and 10 plasma protein candidates among three groups

	HC (100)	NSCLC (110)	BPD (108)	HC_vs_NSCLC	NSCLC_vs_ BPD	HC_vs_BPD
**Ala**	492.48(470.74 ~ 514.22)	508.45(486.29 ~ 530.60)	459.63(437.66 ~ 481.60)	0.310	0.002	0.037
**Arg**	119.39(113.92 ~ 124.87)	117.54(110.41 ~ 124.67)	113.34(106.91 ~ 119.76)	0.509	0.206	0.034
**Cit**	31.59(29.72 ~ 33.47)	38.46(35.87 ~ 41.05)	29.80(28.00 ~ 31.60)	< 0.001	< 0.001	0.112
**Glu**	232.27(221.71 ~ 242.84)	249.11(232.54 ~ 265.69)	232.32(218.96 ~ 245.68)	0.671	0.350	0.826
**Gly**	328.17(316.79 ~ 339.55)	349.13(332.02 ~ 366.23)	335.82(317.63 ~ 354.01)	0.369	0.259	0.780
**His**	102.51(99.16 ~ 105.86)	88.67(85.54 ~ 91.80)	83.71(80.55 ~ 86.88)	< 0.001	0.044	< 0.001
**Leu**	176.86(168.99 ~ 184.74)	173.87(166.79 ~ 180.95)	165.40(158.78 ~ 172.03)	0.309	0.153	0.022
**Lys**	280.72(268.26 ~ 293.18)	253.89(243.85 ~ 263.93)	242.94(232.12 ~ 253.76)	< 0.001	0.087	< 0.001
**Orn**	131.70(122.28 ~ 141.12)	152.05(143.48 ~ 160.61)	149.18(139.19 ~ 159.18)	< 0.001	0.464	0.005
**Met**	28.79(27.85 ~ 29.72)	30.28(28.96 ~ 31.60)	29.41(27.95 ~ 30.87)	0.315	0.364	0.936
**Pro**	198.68(186.09 ~ 211.27)	211.03(198.16 ~ 223.89)	195.82(185.94 ~ 205.70)	0.420	0.302	0.983
**Phe**	103.83(97.88 ~ 109.77)	106.27(101.71 ~ 110.82)	106.44(101.66 ~ 111.226)	0.320	0.614	0.590
**Tyr**	82.00(79.05 ~ 84.95)	77.60(74.42 ~ 80.77)	76.62(73.57 ~ 79.67)	0.047	0.662	0.013
**Val**	287.63(275.91 ~ 299.36)	307.66(296.48 ~ 318.84)	275.65(263.98 ~ 287.32)	0.015	< 0.001	0.153
**CA**	189.99(109.76 ~ 270.22)	493.07(273.37 ~ 712.78)	181.73(57.98 ~ 305.48)	0.011	0.001	0.508
**DCA**	432.71(351.24 ~ 514.18)	311.21(227.11 ~ 395.31)	139.50(85.36 ~ 193.64)	0.004	< 0.001	< 0.001
**CDCA**	1021.85(789.84 ~ 1253.87)	1360.35(918.62 ~ 1802.08)	789.31(539.28 ~ 1039.35)	0.953	0.006	0.005
**UDCA**	257.42(201.39 ~ 313.45)	190.33(139.66 ~ 241.00)	146.59(90.32 ~ 202.85)	< 0.001	0.002	< 0.001
**LCA**	24.70(20.19 ~ 29.22)	14.68(9.22 ~ 20.14)	11.09(8.84 ~ 13.35)	< 0.001	0.922	< 0.001
**GCA**	278.70(221.78 ~ 335.61)	423.39(258.99 ~ 587.79)	348.50(261.19 ~ 435.81)	0.958	0.942	0.870
**GLCA**	10.19(7.41 ~ 12.96)	7.94(5.46 ~ 10.42)	4.85(2.44 ~ 7.26)	0.033	0.052	< 0.001
**GDCA**	223.27(170.36 ~ 276.18)	417.09(239.57 ~ 594.62)	101.05(67.78 ~ 134.32)	0.593	< 0.001	< 0.001
**GCDCA**	871.95(742.94 ~ 1000.95)	1755.82(1386.13 ~ 2125.50)	1604.76(1325.65 ~ 1883.88)	< 0.001	0.914	< 0.001
**GUDCA**	268.93(217.68 ~ 320.19)	372.22(292.75 ~ 451.70)	428.74(250.72 ~ 606.76)	0.128	0.026	0.372
**TCA**	36.42(22.86 ~ 49.98)	60.60(29.70 ~ 91.51)	83.72(37.85 ~ 129.60)	0.758	0.166	0.077
**TLCA**	1.12(0.87 ~ 1.37)	1.43(0.71 ~ 2.14)	0.65(0.40 ~ 0.89)	0.106	0.006	< 0.001
**TDCA**	76.54(63.94 ~ 89.13)	82.53(49.93 ~ 115.12)	32.79(24.47 ~ 41.11)	< 0.001	0.001	< 0.001
**TCDCA**	105.18(86.12 ~ 124.24)	202.43(142.35 ~ 262.50)	290.78(205.57 ~ 375.99)	0.062	0.016	< 0.001
**TUDCA**	13.28(4.17 ~ 22.40)	8.20(6.22 ~ 10.18)	14.01(9.54 ~ 18.47)	0.674	0.596	0.544
**CA125**	14.12(12.56 ~ 15.68)	25.97(18.37 ~ 33.57)	21.56(17.16 ~ 25.97)	0.691	0.104	0.015
**CA199**	13.17(11.31 ~ 15.03)	27.76(17.94 ~ 37.58)	16.41(9.67 ~ 23.14)	0.019	0.004	0.627
**CEA**	1.96(1.68 ~ 2.24)	11.46(4.70 ~ 18.23)	2.89(2.37 ~ 3.41)	< 0.001	0.007	< 0.001
**CYFRA211**	2.18(1.96 ~ 2.40)	5.92(3.68 ~ 8.16)	3.06(2.65 ~ 3.48)	< 0.001	0.013	0.001
**NSE**	13.03(12.43 ~ 13.62)	12.91(11.95 ~ 13.86)	10.89(10.21 ~ 11.56)	0.107	< 0.001	< 0.001
**SCC**	1.41(1.26 ~ 1.56)	2.75(1.30 ~ 4.20)	1.41(1.12 ~ 1.70)	0.918	1.120	0.103
**ApoA1**	1.49(1.44 ~ 1.53)	1.36(1.32 ~ 1.41)	1.13(1.07 ~ 1.20)	< 0.001	< 0.001	< 0.001
**ApoA2**	25.76(25.12 ~ 26.40)	22.57(21.81 ~ 23.32)	18.30(17.24 ~ 19.37)	< 0.001	< 0.001	< 0.001
**ApoB**	0.84(0.81 ~ 0.87)	0.93(0.89 ~ 0.98)	0.83(0.78 ~ 0.87)	0.001	< 0.001	0.613
**ApoC3**	10.26(9.68 ~ 10.85)	10.30(9.58 ~ 11.02)	9.16(8.32 ~ 10.00)	0.719	0.011	0.003
**C1INH**	0.29(0.28 ~ 0.19)	0.32(0.31 ~ 0.33)	0.32(0.30 ~ 0.33)	< 0.001	0.661	0.001
**C3**	1.13(1.08 ~ 1.18)	1.26(1.21 ~ 1.32)	1.15(1.10 ~ 1.21)	< 0.001	0.001	0.960
**C4**	0.24(0.23 ~ 0.25)	0.28(0.26 ~ 0.29)	0.27(0.26 ~ 0.29)	0.005	0.955	0.006
**FN**	548.51(514.84 ~ 582.19)	435.43(401.12 ~ 469.74)	339.23(312.41 ~ 366.05)	< 0.001	< 0.001	< 0.001
**Lpa**	225.25(172.84 ~ 277.66)	298.87(242.88 ~ 354.87)	228.29(186.28 ~ 270.30)	0.060	0.047	0.928
**Fg**	2.66(2.55 ~ 2.77)	3.38(3.14 ~ 3.61)	3.93(3.62 ~ 4.25)	< 0.001	0.019	< 0.001

The concentration units of these candidates is in supplementary Table 10

Compared with BPD group, the plasma level of Fg in the NSCLC group was significantly lower (*P <* 0.05), while the levels of ApoA1, ApoA2, ApoB, ApoC3, C3, FN, and Lp(a) were higher (*P <* 0.05) (Table 2, Supplementary Fig. 2). The Ala, His, Val, and Cit in the NSCLC group all increased (*P <* 0.05) (Supplementary Fig. 3). The TCDCA of the NSCLC group decreased (*P <* 0.05), whereas the CA, CDCA, DCA, UDCA, GDCA, GLCA, GUDCA, TDCA, and TLCA increased (*P <* 0.05) (Supplementary Fig. 4). The CA19-9, CEA, CYFRA21-1, and NSE in the NSCLC group were significantly higher than those in the BPD group (*P <* 0.05), and there was no significant difference in CA12-5 and SCC between the two groups (*P >* 0.05) (Supplementary Fig. 5).

### The highest performance of candidate features surpasses those of classic tumor biomarkers

**Fig. 3:**
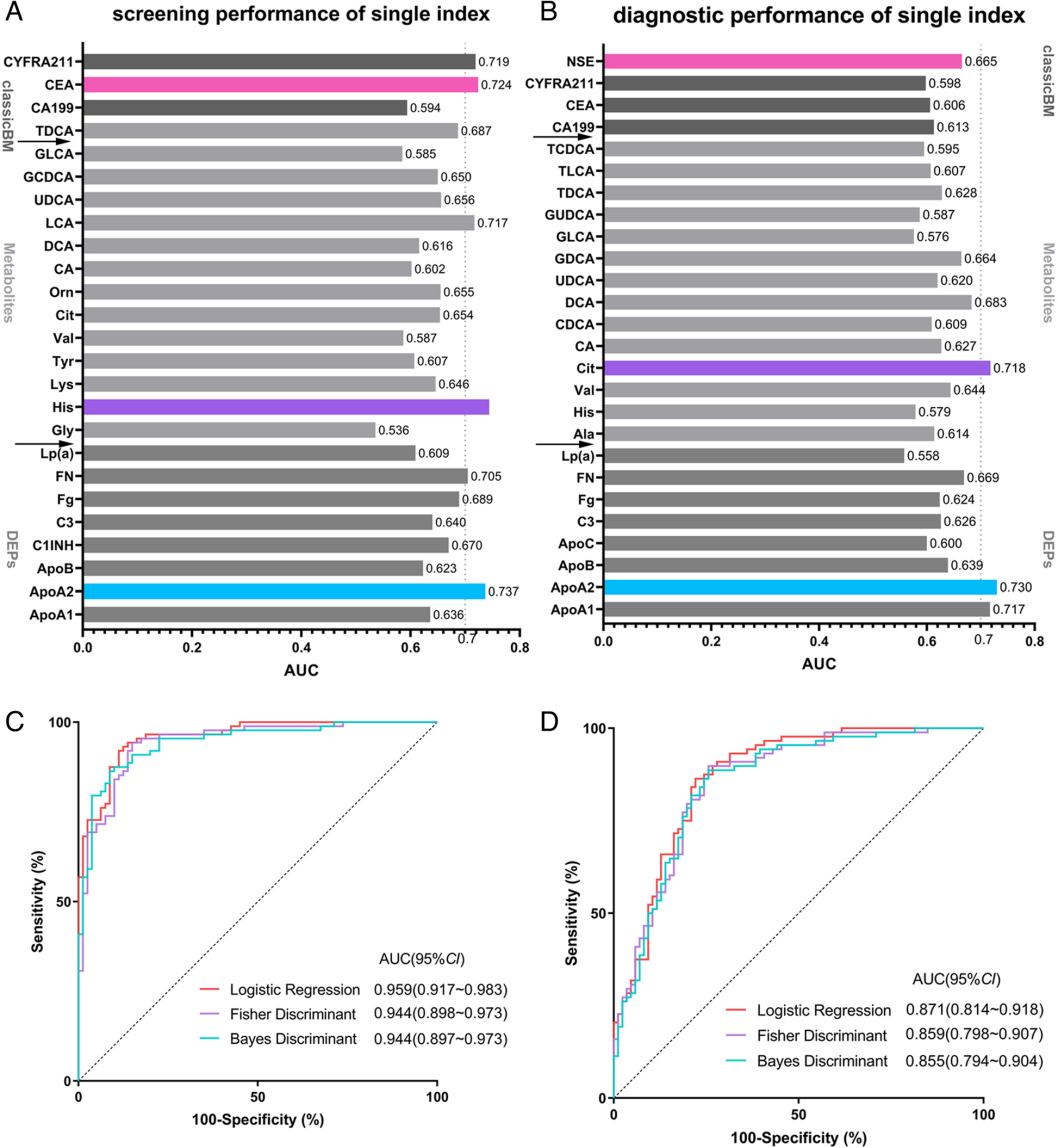
Performance of single index and three supervised learning algorithms comparison.** A** The best single index for screening NSCLC is His, which AUC is 0.744. **B** The best single index for diagnosing NSCLC is ApoA2, which AUC is 0.730. **C** The logistic regression is with the best performance for screening NSCLC among three models. **D** The logistic regression is with the best performance for diagnosing NSCLC among three models

For NSCLC screening, we performed receiver operating characteristic curve (ROC) analyses. We found that His is the best single predictor with area under curve (AUC) of 0.744, sensitivity of 66%, specificity of 79%; and the following is ApoA2, with AUC of 0.737, sensitivity of 59%, 82% of specificity. His and ApoA2 presented a higher AUC than that of the best tumor biomarker CEA, with AUC of 0.724, sensitivity of 59%, and specificity of 74% (Fig. 3A).

For NSCLC diagnosis, we found ApoA2 is the best single predictor, with AUC of 0.730, sensitivity of 86%, and specificity of 56%, and following is Cit, with AUC of 0.718, sensitivity of 83%, and specificity of 57%. ApoA2 and Cit showed better performance than the best tumor biomarker NSE for diagnosis (Fig. 3B). Therefore, the highest performance of candidate features in metabolite and protein group was better than those of classical tumor biomarkers.

### Machine learning algorithms and model selection

To identify high-performance panels for lung cancer screening and diagnosis, we applied stepwise binary logistic regression, stepwise Fisher discriminant, and stepwise Bayes discriminant to optimize our models. Twenty-five candidate features with differences between NSCLC and HC groups were inputted into screening model, and 26 features with differences between BPD and NSCLC groups were inputted into diagnostic model. The features whose signs in the model did not agree with an upward or downward trend in clinical assessments were removed. After that, the initiation of integrated screening model consists of 19 variables, including ApoA2, ApoB, C3, C4, Fg, FN, Gly, CA19-9, CYFRA21-1, His, Lys, Cit, Orn, CA, DCA, LCA, UDCA, GCDCA, and CEA, and integrated diagnostic model initiates with 20 variables, including ApoA1, ApoA2, ApoB, ApoC3, Lp(a), C3, FN, Fg, Ala, His, Val, Cit, CA, CDCA, GDCA, TCDCA, CA19-9, CEA, CYFRA21-1, and NSE. Backward elimination was individually performed in all three algorithms to analyze and optimize potential predictors. We randomly apportion the data into training and test sets in a ratio of 80:20. The *P* value < 0.05 was regarded as significant.

In detail, 80% of the sample size was randomly selected to form training samples, with 88 cases in the NSCLC group and 80 cases in the HC group, and 86 cases in the BPD group. Three machine learning algorithms, stepwise logistic regression, stepwise Fisher discriminant, and stepwise Bayes discriminant, were applied to build the screening and diagnosis model. Three optimized screening models using different algorithms, and four additional screening models with different predictors were built. For differential diagnosis, there are also three models different in algorithms and four additional models with different indicators. The backward stepwise selection was used in model building process to filter out variables with lower weight. Variables with *P* value > 0.05 or Wald value < 1 were removed to obtain a high-performance model. We specially tested the binary logistic regression models. In 12 cycles of elimination, we removed DCA, C4, Fg, Lys, CYFRA21-1, CA19-9, and Gly sequentially. Finally, 12 corresponding models were built, and the performance was predicted using ROC and Youden index analyses. Similar analyses were performed when building diagnostic models. In 17 cycles of selection, ApoB, ApoA1, His, TDCA, CA19-9, GUDCA, UDCA, CA, Ala, Val, FN, ApoC3, CEA, CDCA, NSE, and Fg were removed sequentially, and corresponding models were built based on ROC and Youden index analyses. The change of AUC and Youden index values by steps of elimination was shown in line charts (Supplementary Fig. 9). According to the significance and Youden index, the 8th screening model and the 15th diagnostic model were considered as the best models.

The remaining 20% samples were made up of 22 NSCLC cases, 20 HC cases, and 22 BPD cases. We used these samples to evaluate the models. The case values were substituted into models, with results higher than the optimal threshold judged as NSCLC cases. If the results were lower than the optimal threshold, the cases would be judged as HC cases in screening model and BPD cases in diagnosis model (Supplementary Tables 5 and 8). The coincidence rate of the logistic regression model was higher than that of Fisher discriminant model and Bayes discriminant model, either in screening or diagnostic model (Fig. 3C and D). The evaluation performance showed significant similarity with predictors, which indicated the binary logistic regression models were stable and were the best choice for screening and diagnosis (Supplementary Tables 6 and 9). The formula is described in the methods.

Finally, we obtained the most high-performance screening model and diagnostic model by logistic regression. The screening model has 12 signatures, consisting of ApoA2, ApoB, C3, FN, His, Cit, Orn, CA, UDCA, LCA, GCDCA, CEA. The screening model has an AUC of 0.959 (95%CI: 0.917 ~ 0.983) with sensitivity of 92% and specificity of 89%. The diagnostic model has 9 signatures, consisting of ApoA2, Lp(a), C3, Fg, Cit, GDCA, TCDCA, CYFRA21-1, NSE. The diagnostic model has an AUC of 0.871(95%CI: 0.814 ~ 0.918), with sensitivity of 86% and specificity of 78%.

### Validation of screening and diagnostic panel

**Fig. 4:**
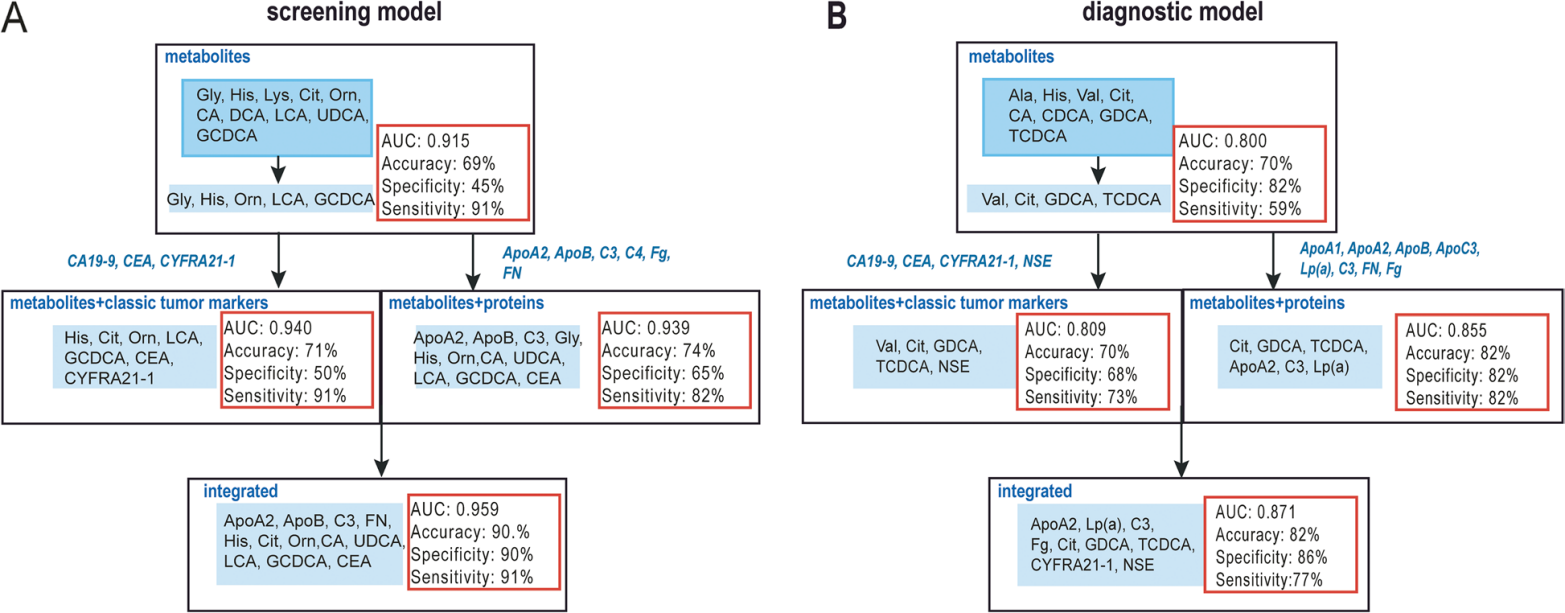
The screening and diagnostic model performance from single omics to multi-omics.** A** Integrated screening model superior to single omics models. **B** Integrated diagnostic model superior to single omics models

We evaluated the performance of models using data of the test set. The six proteins (ApoA2, ApoB, C3, C4, Fg, FN) with significant differences between NSCLC and HC groups were selected to build single omics model for screening. The optimized model consisted of 5 proteins (ApoA2, ApoB, C3, FN, Fg). The AUC value is 0.796 (95%CI: 0.727 ~ 0.854), with accuracy of 83%, sensitivity of 68%, and specificity of 100%. We also selected 10 small-molecule metabolites (Gly, His, Lys, Cit, Orn, CA, DCA, LCA, UDCA, GCDCA) with significant differences between NSCLC and HC groups to build the metabolite model for screening. The final model consisted of 5 small-molecule metabolites (Gly, His, Orn, LCA, GCDCA). The AUC value is 0.915 (95%CI: 0.826 ~ 0.952), with accuracy of 69%, sensitivity of 91% and specificity of 45%. Compared with single biomarker or DEP panel model, the metabolite model shows higher performance power for screening. After that, we added 3 classic tumor markers (CA19-9, CEA, CYFRA21-1) to the metabolite model, and tried to increase the AUC of the model. And the final model consists of 7 variables (His, Cit, Orn, LCA, GCDCA, CEA, CYFRA21-1). The AUC of the model is 0.940 (95%CI: 0.893 ~ 0.971), with accuracy of 71%, sensitivity of 91% and specificity of 50%. While we added 6 DEPs (ApoA2, ApoB, C3, C4, Fg, FN) to 10 small-molecule metabolites, the AUC of the model is 0.939 (95%CI: 0.891 ~ 0.970) with accuracy of 74%, sensitivity of 82%, and specificity of 65%. The all-integrated final model consisted of 12 biomarkers (ApoA2, ApoB, C3, FN, His, Cit, Orn, CA, UDCA, LCA, GCDCA, CEA), with further increased AUC of 0.959 (95%CI: 0.917 ~ 0.983), accuracy of 90%, sensitivity of 91%, and specificity of 90%, which achieved significant improvement over the original single-omics model (Fig. 4A).

When building the diagnostic model, we selected 8 proteins (ApoA1, ApoA2, ApoB, ApoC3, Lp(a), C3, FN, Fg) to build the protein model. We obtained a final protein model with AUC of 0.750 (95%CI: 0.679 ~ 0.812), accuracy of 80%, sensitivity of 76%, and specificity of 73%. We also selected 8 small-molecule metabolites (Ala, His, Val, Cit, CA, CDCA, GDCA, TCDCA) to build the model, then obtained single omics model with 4 variables (Val, Cit, GDCA, TCDCA). The resulting model had AUC of 0.800 (95%CI: 0.733 ~ 0.857) with accuracy of 70%, sensitivity of 59%, and specificity of 82% (Fig. 4B). Compared with the protein model, metabolite model has better performance. Based on this, the addition of classic tumor biomarkers (CA19-9, CEA, CYFRA21-1, NSE) for backward elimination modeling ended up with a 5-biomarker model (Val, Cit, GDCA, TCDCA, NSE). The model achieved AUC of 0.809 (95%CI: 0.743 ~ 0.857) with accuracy of 70%, sensitivity of 73%, and specificity of 68%. Modeling with small-molecule metabolites and DEPs in NSCLC and BPD groups (ApoA1, ApoA2, ApoB, ApoC3, Lp(a), C3, FN, Fg), ended up with a 6-variable model (Cit, GDCA, TCDCA, ApoA2, C3, Lp(a)). The AUC is 0.855 (95%CI: 0.794 ~ 0.904) with accuracy of 82%, sensitivity of 82%, and specificity of 82%. Adding classic tumor biomarkers (CA19-9, CEA, CYFRA21-1), then the final model consisted of 9 variables (ApoA2, Lp(a), C3, Fg, Cit, GDCA, TCDCA, CYFRA21-1, NSE), and the AUC increased to 0.871 (95%CI: 0.814 ~ 0.918) with accuracy of 81.82%, sensitivity of 77%, and specificity of 86%.

Our results showed that the integrated model features perform better than those of single omics or classic tumor biomarker features alone. In test set, the screening panel has an accuracy of 90%, specificity of 90%, and sensitivity of 91%. The diagnostic panel has an accuracy of 82%, sensitivity of 77%, and specificity of 86%.

## Discussion

In this study, we tried to establish novel integrated detection models for early and non-invasive screening and accurately differential diagnosis of NSCLC based on multi-omics together with machine learning algorithms. Moreover, we provided an approach to detect DEPs, amino acids, bile acids, and classic tumor biomarkers in blood samples for non-invasive detection of cancer.

Improved earlier cancer detection can make cancer treatment more effective, and survival improves dramatically [16]. As clinical classic biomarkers, CA12-5, CA19-9, CEA, CYFRA21-1, NSE, and SCC, are the most commonly used for screening and differential diagnosis of lung cancer. Generally, the sensitivity of one of these indicators applied alone is 30–50%, and the specificity is 45–70%. The incorporation of these classic biomarkers can improve accuracy, and the sensitivity and specificity of the panels of these indexes were increased to about 85% and 80% respectively. Compared to the clinical and health examination requirements, the accuracy of these tests has yet to be improved [17, 18]. Recently, more and more multi-omics integration technologies are applied to discover all kinds of markers for tumor screening, prognosis [19], and personalized treatment [20, 21]. Some studies about multi-omics, especially genomics and transcriptomics analysis of tissues to explore the pathogenesis and novel treatment strategy for lung cancer have been reported [22–25]. However, inheriting cancer-predisposing nucleic acid alterations does not always lead to the development of the disease [26]. By integrating proteomics and metabolomics, we can not only understand the biological process but also discover predictors of disease or disease outcome. For example, integrated proteomics and metabolomics approaches in blood samples help us understand the development of coronavirus disease 2019 [27] and breast cancer [28], and accelerate the discovery of biomarkers for the detection of colorectal cancer [29] and pancreatic cancer [30]. However, although there are some research works on the biomarkers of for lung cancer through single omics, there are few reports on the joint identification of biomarkers through proteomics and metabolomics for lung cancer detection in blood samples [12, 31, 32]. By blood proteomic analysis, researchers found that AMBP, α_2_ macroglobulin, and SERPINA1 might be valuable biomarkers for early detection of lung cancer using serum from 20 NSCLC and 10 healthy donors [33], and it would be better to perform a clinical validation in a larger cohort. Some research has both discovery set and validation set [34, 35], and if they added a BPD group, such as cases of pulmonary nodules or tuberculosis granuloma, their diagnostic performance would be more reliable. In lung cancer metabolomics research, the partial least squares-discriminant analysis (PLS-DA) model using glycine, valine, methionine, citrulline, arginine, and C16-carnitine, and they were validated in 40 lung cancer patients and 100 controls [36]. Another research obtained six metabolic biomarkers by combing metabolomics and machine learning methods [37]. And by using machine learning methods, these metabolomics research obtained high performance panels in lung cancer screening, but most lung cancer blood-based biomarkers reported are involved in a single class panel, and the integrative research of lung cancer based on plasma protein and metabolites is deficient [38, 39]. In this study, we unveiled that the complement and coagulation cascade system is aberrantly active, and amino acids and lipid metabolism were disturbed in blood of treatment naïve NSCLC patients. Hypercoagulation [40] and complement [41] can facilitate lung cancer development and metastasis. Tumor rewires amino acids metabolism to regulate anti-tumor immune response in the tumor microenvironment [42] and promote cancer proliferation [43]. However, the reason why plasma His is low and plasma Cit and Orn are high in NSCLC remains to be further explored. Cancer cells can disrupt lipid metabolism to promote tumor growth and dissemination [44]. Bile acids play a nonnegligible role in carcinogenesis [45]. For example, UDCA shows anti-tumor ability, while DCA promotes lung cancer cell growth, migration, and invasion [46]. Most studies focused on bile acid metabolism in digestive tract diseases [47], gastrointestinal and liver cancer [48]. However, the functional mechanism of bile acid metabolism in NSCLC remains to be further studied.

With the improvement of mass spectrometry technology and omics, and the development of artificial intelligence algorithms, it is possible to screen specific markers or combinations of markers from abnormal disease metabolism [29, 30, 33, 34]. This study introduced advanced TMT-LC/MS technology and bioinformatics, and obtained the efficient integrative detection panels based on plasma proteomics-guided metabolite profiling. The candidate biomarkers, including complement coagulation protein, amino acid, and bile acid molecules in blood plasma were evaluated with commercial kits. Three machine learning algorithms were used to integrate and analyze the detection panels. As far as we know, this is the first time to systematically reveal the close relationship between lung cancer and complement coagulation molecules, non-protein amino acids involved in urea metabolism, and primary bile acids and secondary bile acids related to bile acid metabolism. Our results showed that the integrated model features perform better than single omics or classic tumor biomarker features alone. Actually, proteins and metabolites interact closely in living organisms. On the one hand, proteins can affect the characteristics of metabolites, and on the other hand, metabolites can also affect protein levels through enzymatic reactions. Integrated analysis of proteome and metabolites can provide a more comprehensive understanding of tumorigenesis and aberrant expression of tumor-associated genes and their metabolic products [49]. Therefore, this study not only provided a more accurate detection model for lung cancer screening and diagnosis, but also provided a research direction for further revealing the pathogenesis of lung cancer.

This study had some limitations to consider. First, the new models in a cohort that was mainly composed of untreated patients with NSCLC, which is mainly suitable for early-stage lung cancer screening and differential diagnosis. However, the establishment of models to predict the recurrence, metastasis, and drug resistance of lung cancer may require additional samples and further research. Next, we developed binary classification models. It would be better to build a machine learning model for ternary classification with cross-validation to simplify and improve our NSCLC screening and diagnosis model. Finally, an external test cohort and multicenter clinical evaluation will be better for further validation of the model.

To sum up, we provided the new integrated models for screening and differential diagnosis of NSCLC, which consisted of proteins, non-protein amino acids, and bile acids, and showed better performance compared with classical clinical biomarkers alone. This study also opened up new perspectives for the investigation of aberrant complement coagulation pathway, cholesterol metabolism, and high Cit level of lung cancer, which may offer a valuable therapeutic clue in the ongoing battle against NSCLC. In addition, the plasma proteomics-guided metabolite profiling analysis has initially shown the important application value in the screening of disease-related biomarkers, and also provides a new strategy for the investigation of other kinds of tumor markers.

## Conclusions

In conclusion, we provide the first predictive model for NSCLC screening and diagnosis that consists of proteins, non-protein amino acids and bile acids, which shows better performance than classical clinical panel alone, and open up new perspectives for the study of aberrant cholesterol metabolism, complement coagulation pathway, and high Cit level of lung cancer, which may offer useful therapeutic clue in the ongoing battle against NSCLC. Moreover, the same screening and diagnostic modeling process can be applied to other tumors.

## Supplementary Information

**Additional file 1: Supplementary Figure 1. **GO Enrichment pathway associated with cellular component, and biological process. **Supplementary Figure 2. **The differentially expression of 10 plasma protein candidates among three groups. **Supplementary Figure 3. **The differentially expression of 14 serum amino acids among three groups. **Supplementary Figure 4. **The differentially expression of 15 bile acids among three groups. **Supplementary Figure 5. **The differentially expression of six classic tumor markers among three groups. **Supplementary Figure 6. **Proteins and amnio acids related to NSCLC stage. **Supplementary Figure 7. **Single index with AUC>0.7 for NSCLC screening. **Supplementary Figure 8. **Single index with AUC>0.7 in differentiating NSCLC and BPD. **Supplementary Figure 9.**The process and the result of binary logistic regression with backward elimination methods. **Supplementary Table 1. **Screened differentially expressed proteins and corresponding validation proteins. **Supplementary Table 2. **Performance of single predictor in NSCLC screening. **Supplementary Table 3. **Performance of single predictor in NSCLC diagnosis. **Supplementary Table 4. **Screening model by stepwise binary logistic regression analysis in training samples. **Supplementary Table 5. **Performance analysis of 3 models in screening NSCLC. **Supplementary Table 6. **Testing of 3 models in screening NSCLC. **Supplementary Table 7. **Diagnosis model by stepwise binary logistic regression analysis in training samples. **Supplementary Table 8. **Performance analysis of 3 models in differentiating NSCLC and BPD. **Supplementary Table 9. **Testing of 3 models in differentiating NSCLC and BPD. **Supplementary Table 10. **The concentration units of these candidates.

## Data Availability

The blood samples are collected from 318 patients in Shanghai Jiao Tong University School of Medicine Affiliated Renji Hospital from October 2020 to January 2021, including 110 NSCLC, 100 HC, and 108 BPD patients.

## Abbreviations

NSCLC: Non-small cell lung cancer
TMT-LC-MS/MS: Tandem Mass Tagging-Liquid Chromatography-Tandem Mass Spectrometry
DEPs: Differentially expressed proteins
BPD: Benign pulmonary diseases
HC: Healthy controls
AUC: Area Under the Curve
LDCT: Low-dose computed tomography
GO: Gene Ontology
KEGG: Kyoto Encyclopedia of Genes and Genomes
EDTA: Ethylenediaminetetraacetic acid
SPSS: Statistical Product and Service Solutions
ApoA1: Apolipoprotein A1
ApoA2: Apolipoprotein A2
ApoB: Apolipoprotein B
ApoC3: Apolipoprotein C3
FGA: Fibrinogen alpha chain
FGB: Fibrinogen beta chain
FGG: Fibrinogen gamma chain
Apo(a): Apolipoprotein (a)
C1INH: C1 inhibitor
C3: Complement C3
C4A: Complement C4A
C4B: Complement C4B
FN: Fibronectin
Fg: Fibrinogen
Lp(a): Lipoprotein(a)
Ala: Alanine
Arg: Arginine
Met: Methionine
Glu: Glutamic acid
Gly: Glycine
His: Histidine
Leu: Leucine
Lys: Lysine
Phe: Phenylalanine
Pro: Proline
Tyr: Tyrosine
Val: Valine
Cit: Citrulline
Orn: Ornithine
CA: Cholic acid
CDCA: Chenodeoxycholic acid
DCA: Deoxycholic acid
LCA: Lithocholic acid
UDCA: Ursodeoxycholic acid
GCA: Glycocholic acid
GCDCA: Glycochenodeoxycholic acid
GDCA: Glycodeoxycholic acid
GLCA: Glycolithocholic acid
GUDCA: Glycoursodeoxycholic acid
TCA: Taurocholate acid
TCDCA: Taurochenodeoxycholic acid
TDCA: Tauroursodeoxycholic acid
TLCA: Taurolithocholic acid
TUDCA: Tauroursodeoxycholic acid
SCC: Squamous cell carcinoma antigen
CA12-5: Carbohydrate antigen 12-5
CA19-9: Carbohydrate antigen 19-9
CEA: Carcinoembryonic antigen
CYFRA21-1: Cytokeratin 19 fragment 21-1
NSE: Neuron-Specific Enolase
AMBP: Alpha-1-microglobulin/bikunin precursor
SERPINA1: Serpin Family A Member 1
PLS-DA: Partial least squares-discriminant analysis
