# Supplementary material for: Integrated models of blood protein and metabolite enhance the diagnostic accuracy for Non-Small Cell Lung Cancer
Source: Biomark Res. 2023 Jul 20;11:71. doi: 10.1186/s40364-023-00497-2 (PMC10360339; doi:10.1186/s40364-023-00497-2)
Supplement: Supplementary file 1 — Additional file 1: Supplementary Figure 1. GO Enrichment pathway associated with cellular component, and biological process. Supplementary Figure 2. The differentially expression of 10 plasma protein candidates among three groups. Supplementary Figure 3. The differentially expression of 14 serum amino acids among three groups. Supplementary Figure 4. The differentially expression of 15 bile acids among three groups. Supplementary Figure 5. The differentially expression of six classic tumor markers among three groups. Supplementary Figure 6. Proteins and amnio acids related to NSCLC stage. Supplementary Figure 7. Single index with AUC>0.7 for NSCLC screening. Supplementary Figure 8. Single index with AUC>0.7 in differentiating NSCLC and BPD. Supplementary Figure 9.The process and the result of binary logistic regression with backward elimination methods. Supplementary Table 1. Screened differentially expressed proteins and corresponding validation proteins. Supplementary Table 2. Performance of single predictor in NSCLC screening. Supplementary Table 3. Performance of single predictor in NSCLC diagnosis. Supplementary Table 4. Screening model by stepwise binary logistic regression analysis in training samples. Supplementary Table 5. Performance analysis of 3 models in screening NSCLC. Supplementary Table 6. Testing of 3 models in screening NSCLC. Supplementary Table 7. Diagnosis model by stepwise binary logistic regression analysis in training samples. Supplementary Table 8. Performance analysis of 3 models in differentiating NSCLC and BPD. Supplementary Table 9. Testing of 3 models in differentiating NSCLC and BPD. Supplementary Table 10. The concentration units of these candidates. [file 40364_2023_497_MOESM1_ESM.docx]

**Supplementary Materials**

**Supplementary Figure 1:** GO Enrichment pathway associated with cellular component (A), and biological process (B).

**Supplementary Figure 2:** The differentially expression of 10 plasma protein candidates among three groups.

**Supplementary Figure 3:** The differentially expression of 14 serum amino acids among three groups.

**Supplementary Figure 4:** The differentially expression of 15 bile acids among three groups.

**Supplementary Figure 5:** The differentially expression of six classic tumor markers among three groups.

**Supplementary Figure 6:** Proteins and amino acids related to NSCLC stage.

**Supplementary Figure 7:** Single index with AUC>0.7 for NSCLC screening.

**Supplementary Figure 8:** Single index with AUC>0.7 in differentiating NSCLC and BPD.

**Supplementary Figure 9:** The process and the result of binary logistic regression with backward elimination methods.

**Supplementary Table 1:** Screened differentially expressed proteins and corresponding validation proteins.

**Supplementary Table 2:** Performance of single predictor in NSCLC screening.

**Supplementary Table 3:** Performance of single predictor in NSCLC diagnosis.

**Supplementary Table 4:** Screening model by stepwise binary logistic regression analysis in training samples.

**Supplementary Table 5:** Performance analysis of 3 models in screening NSCLC.

**Supplementary Table 6:** Testing of 3 models in screening NSCLC.

**Supplementary Table 7:** Diagnosis model by stepwise binary logistic regression analysis in training samples.

**Supplementary Table 8:** Performance analysis of 3 models in differentiating NSCLC and BPD.

**Supplementary Table 9:** Testing of 3 models in differentiating NSCLC and BPD.

**Supplementary Table 10:** The concentration units of these candidates.


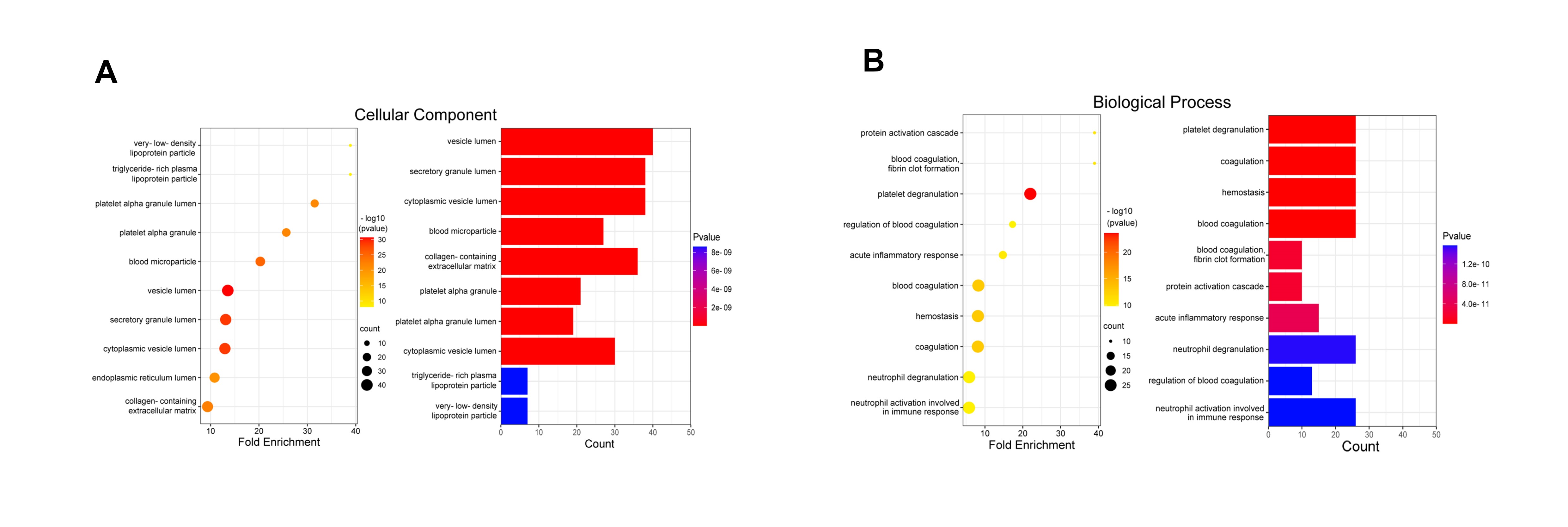


**Supplementary Figure 1:** GO Enrichment pathway associated with cellular component (A), and biological process (B).


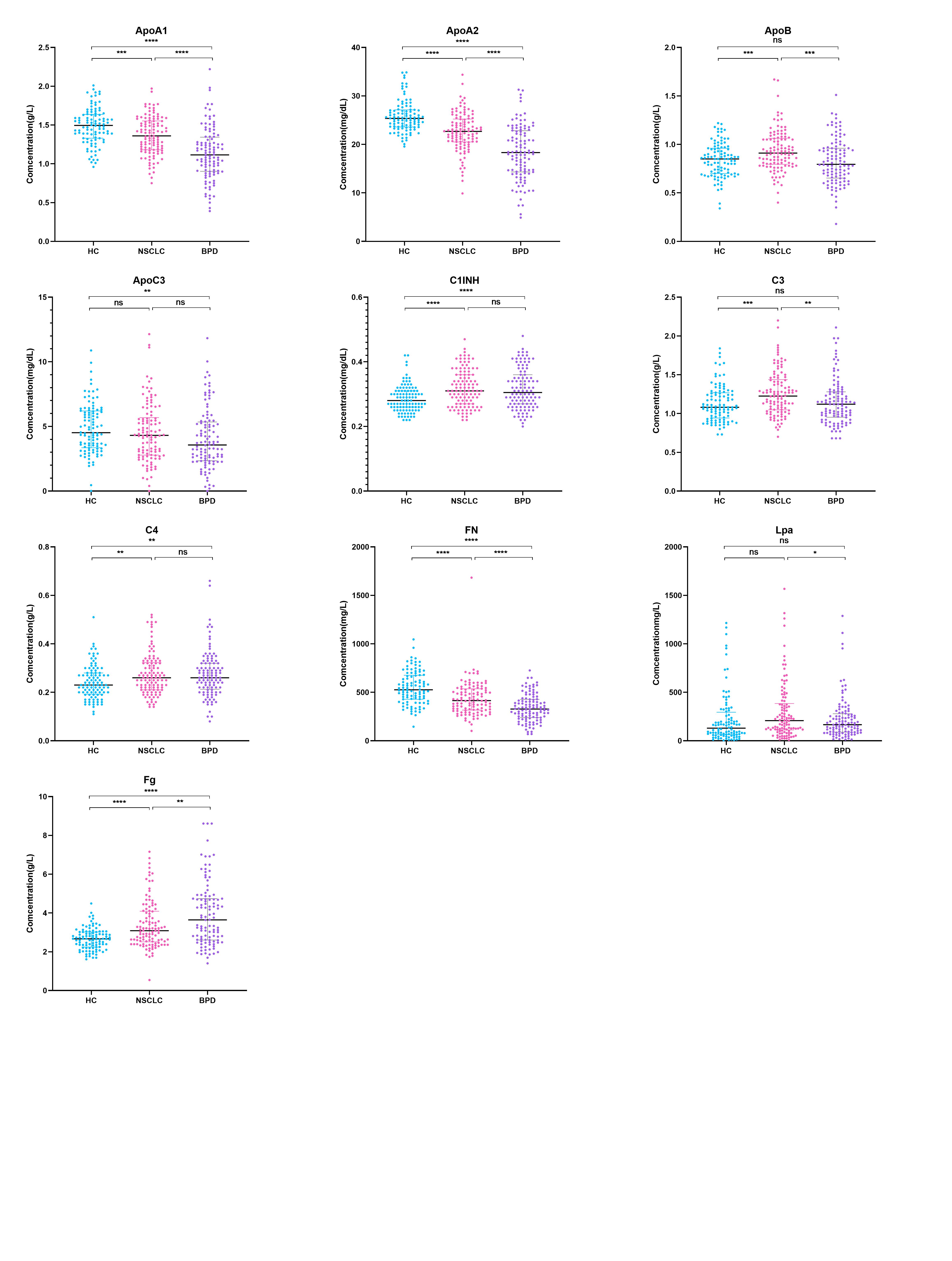


**Supplementary Figure 2:** The differentially expression of 10 plasma protein candidates among three groups. NSCLC vs HC, the plasma level of ApoA1, ApoA2, and FN in the NSCLC group was decreased, while the level of ApoB, C1INH, C3, C4, and Fg was increased, and the difference in plasma ApoC3 concentrations had no statistical significance. NSCLC vs BPD, plasma Fg levels in the NSCLC group was significantly low, while the levels of ApoA1, ApoA2, ApoB, ApoC3, C3, FN, and Lp(a) were high. ns, no significance, *, P<0.05, **, P<0.01, ***, P<0.001, ****, P<0.0001.


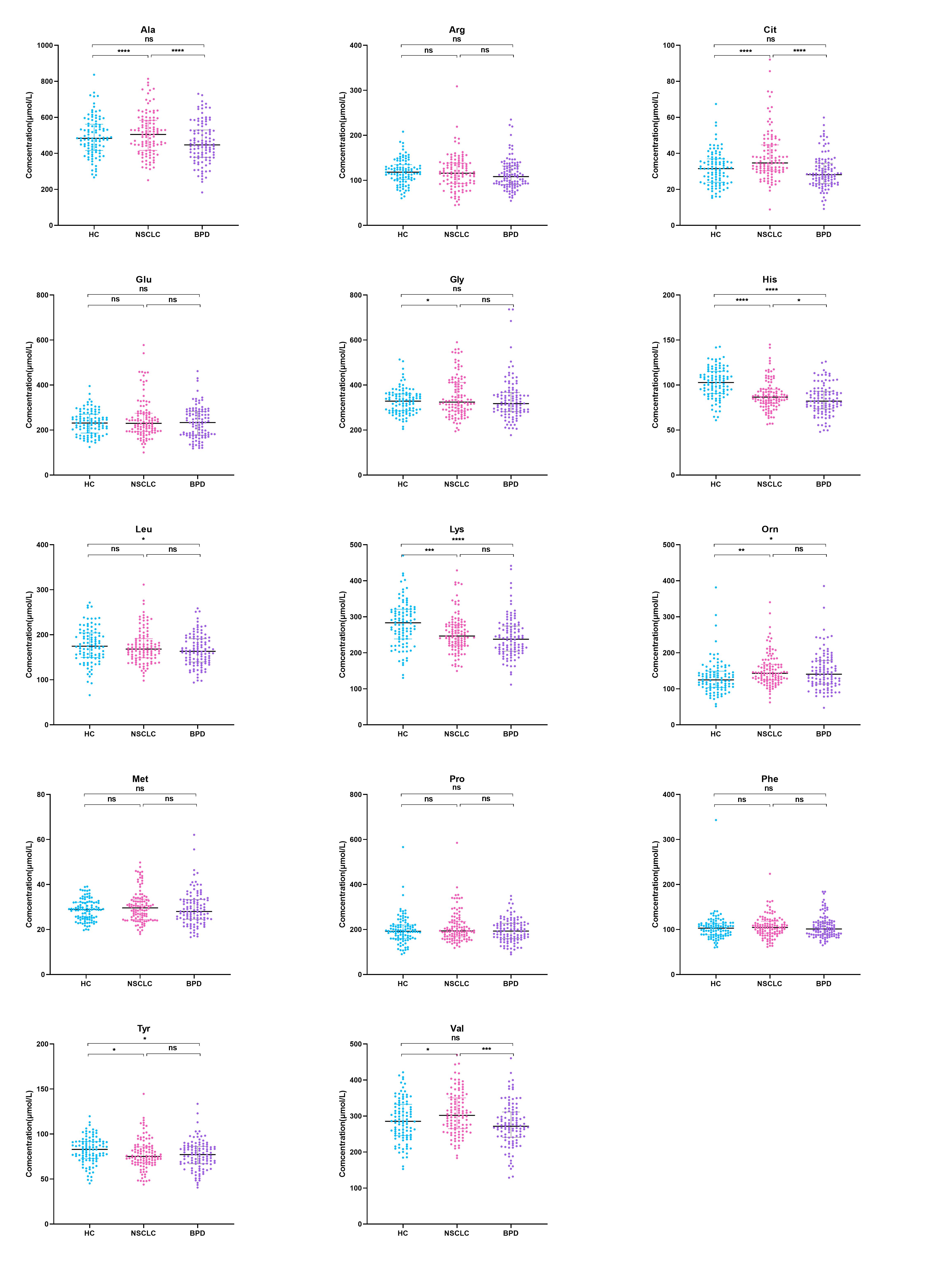


**Supplementary Figure 3:** The differentially expression of 14 serum amino acids among three groups. NSCLC vs HC, the serum His, Lys, Tyr level in the NSCLC group was decreased, and the Gly, Val, Cit, Orn levels increased, while the other serum amino acid concentrations were not significantly different. NSCLC vs BPD, the serum levels of Ala, His, Val, and Cit in the NSCLC group were all increased. ns, no significance, *, *P*<0.05, **, *P*<0.01, ***, *P*<0.001, ****, *P*<0.0001.


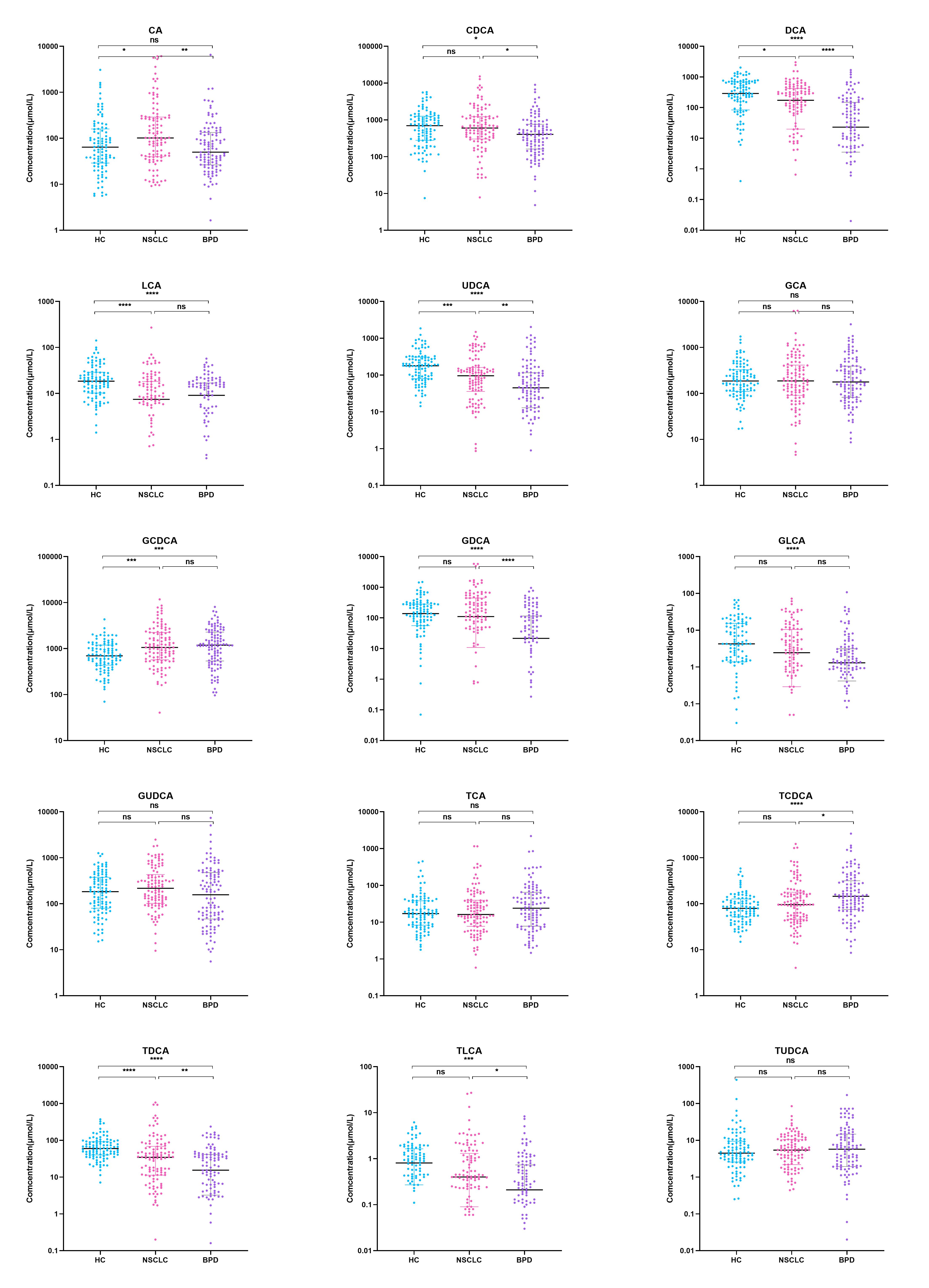


**Supplementary Figure 4:** The differentially expression of 15 bile acids among three groups. NSCLC vs HC, the serum DCA, LCA, UDCA, GLCA, and TDCA levels in the NSCLC group was significantly reduced, and the levels of CA and GCDCA were increased. NSCLC vs BPD, the serum TCDCA levels of the NSCLC group decreased, whereas the levels of CA, CDCA, DCA, UDCA, GDCA, GLCA, GUDCA, TDCA, and TLCA increased. ns, no significance, *, *P*<0.05, **, *P*<0.01, ***, *P*<0.001, ****, *P*<0.0001.


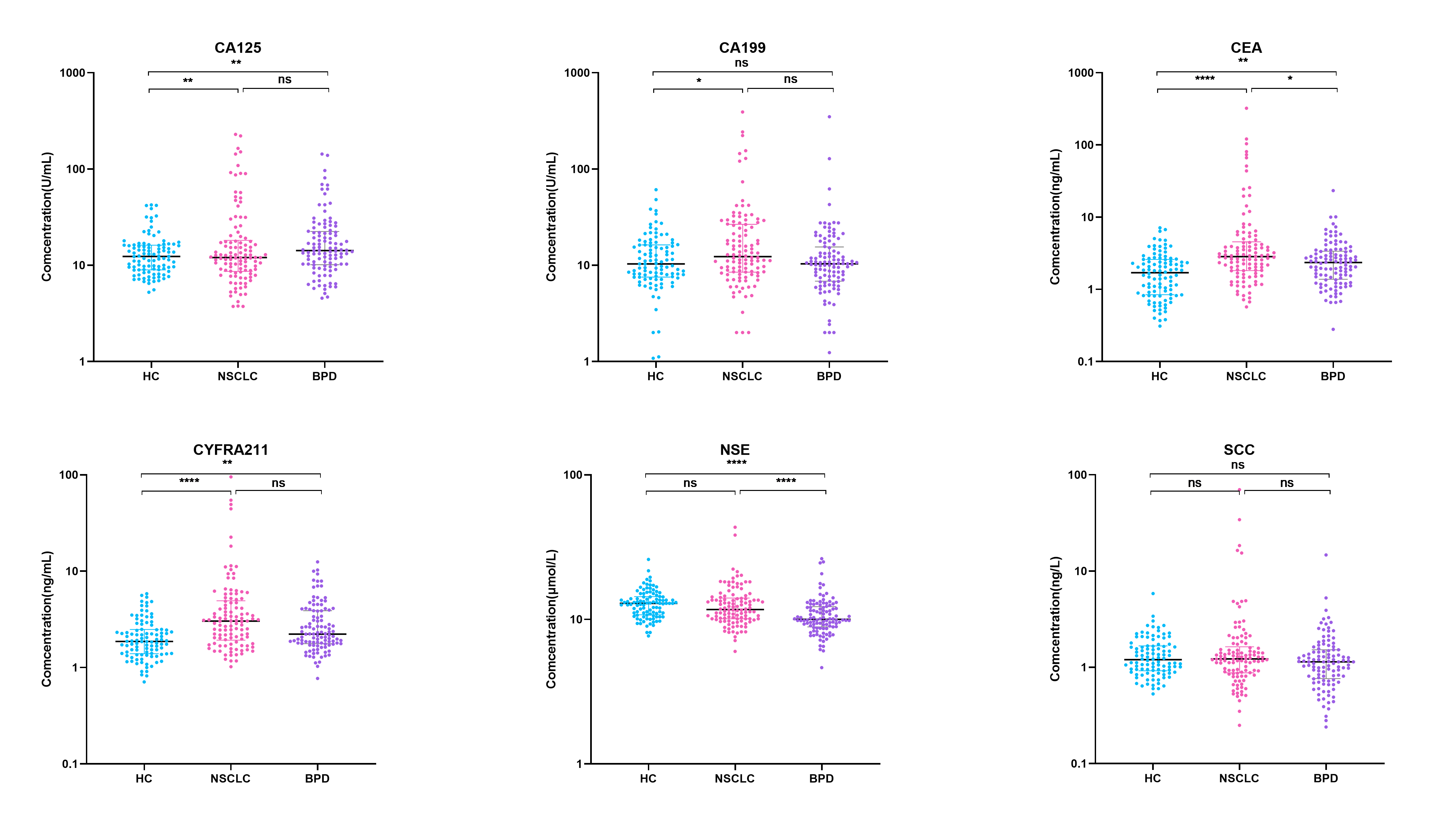


**Supplementary Figure 5:** The differentially expression of six classic tumor markers among three groups. NSCLC vs HC, the serum CA125 levels in the NSCLC group was reduced, and the levels of CA199, CEA and CYFRA211 were increased. NSCLC vs BPD, the serum CEA and NSE levels of the NSCLC group increased. ns, no significance, *, *P*<0.05, **, *P*<0.01, ***, *P*<0.001, ****, *P*<0.0001.


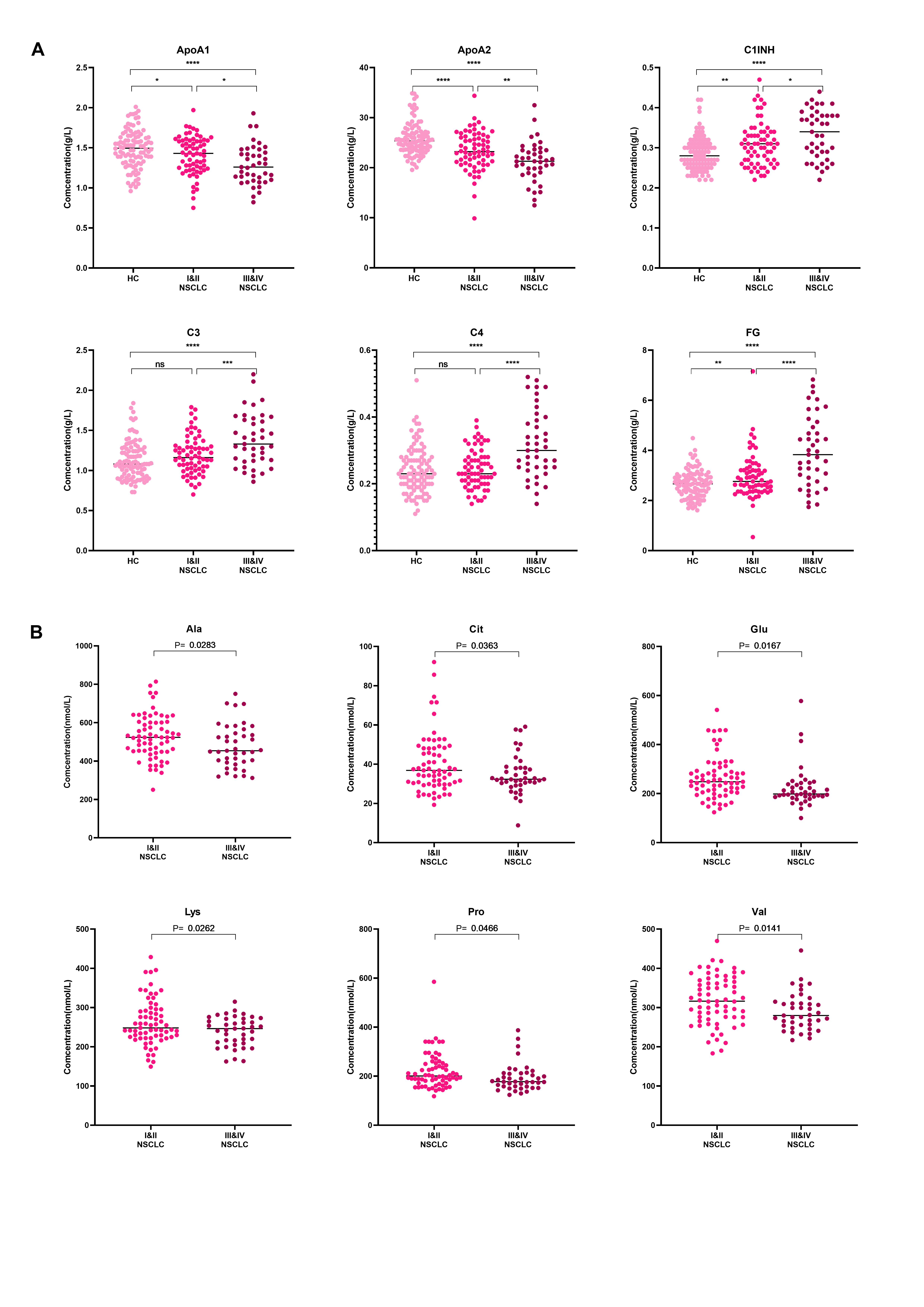


**Supplementary Figure 6: Proteins and amnio acids related to NSCLC stage. NSCLC patients were divided into 67 cases in the early stage (stage I to II) and 41 cases in the middle and late stage (stage III to IV). A.** Plasma ApoA1 and ApoA2 levels gradually decreased with the progression of the disease; C1INH, C3, C4, and Fg gradually increased with the progression of the disease. There was no significant difference between the remaining proteins in the early and middle and late stages of NSCLC. ns, no significance, *, *P*<0.05, **, *P*<0.01, ***, *P*<0.001, ****, *P*<0.0001. **B.** Serum levels of Ala, Glu, Lys, Pro, Val, and Cit gradually decreased with the progression of the disease (*P*<0.05). The remaining small molecule metabolites had no significant difference in the early and middle and late stages of NSCLC.


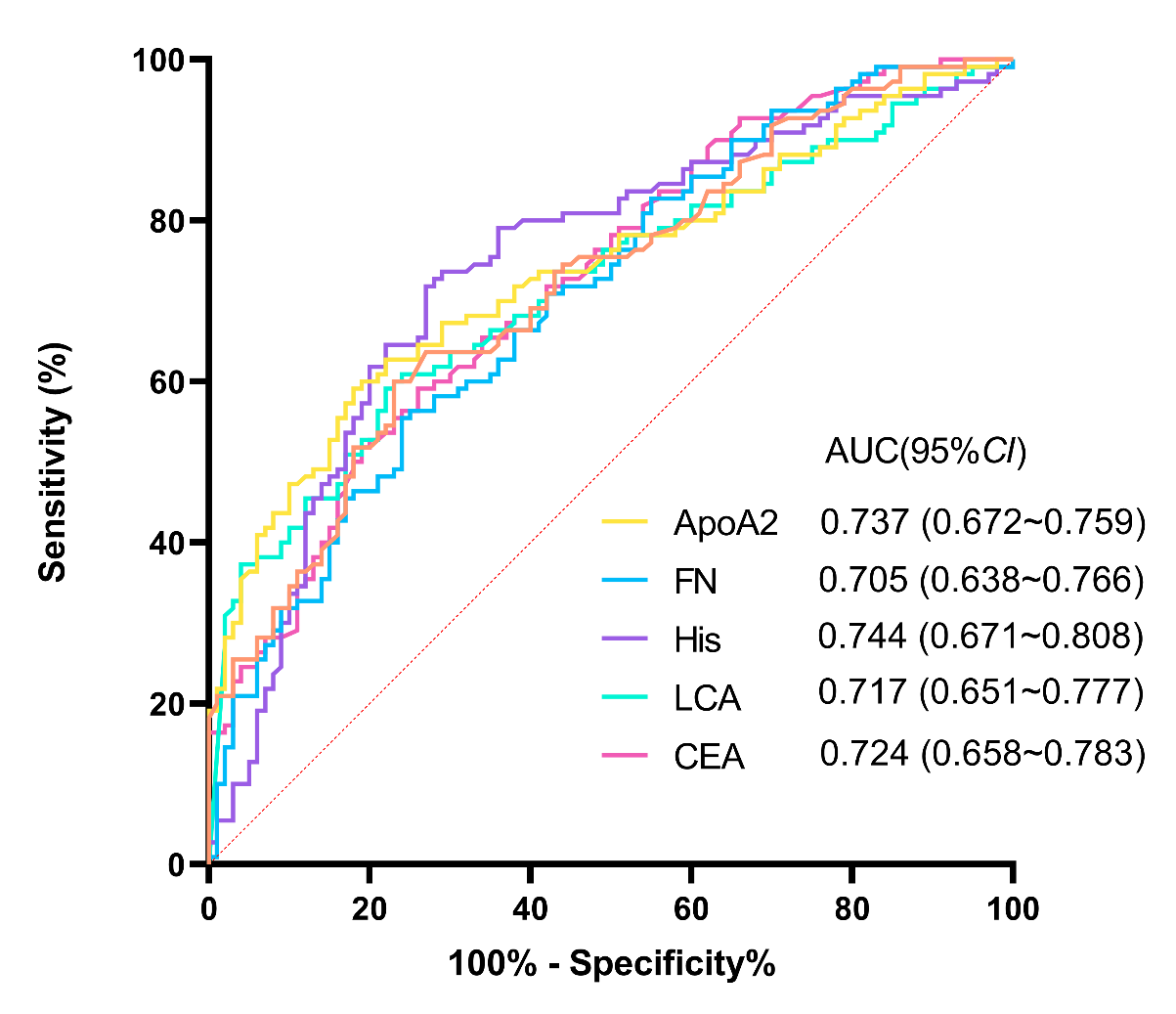


**Supplementary Figure 7:** Single index with AUC>0.7 for NSCLC screening.


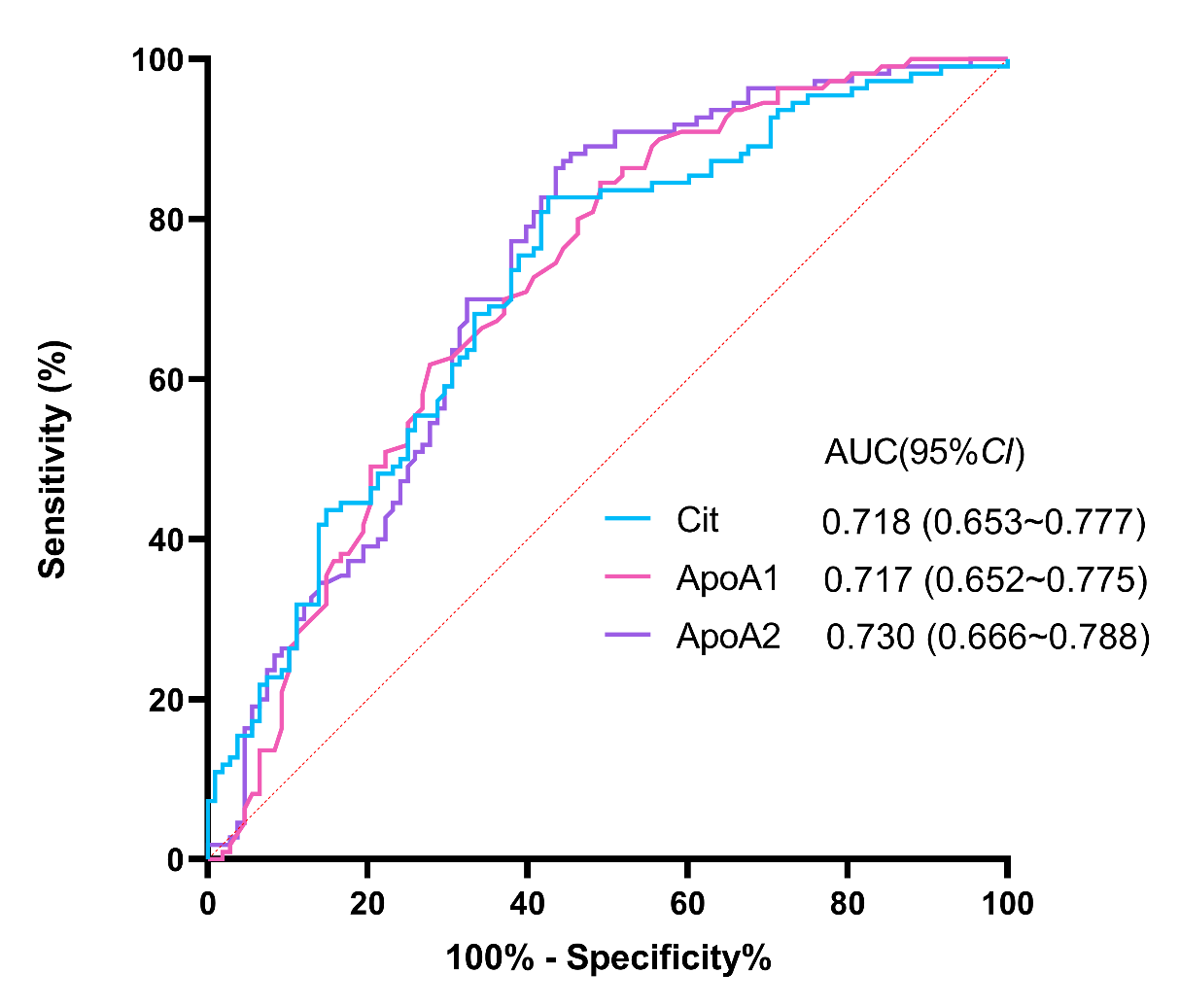


**Supplementary Figure 8:** Single index with AUC>0.7 in differentiating NSCLC and BPD.


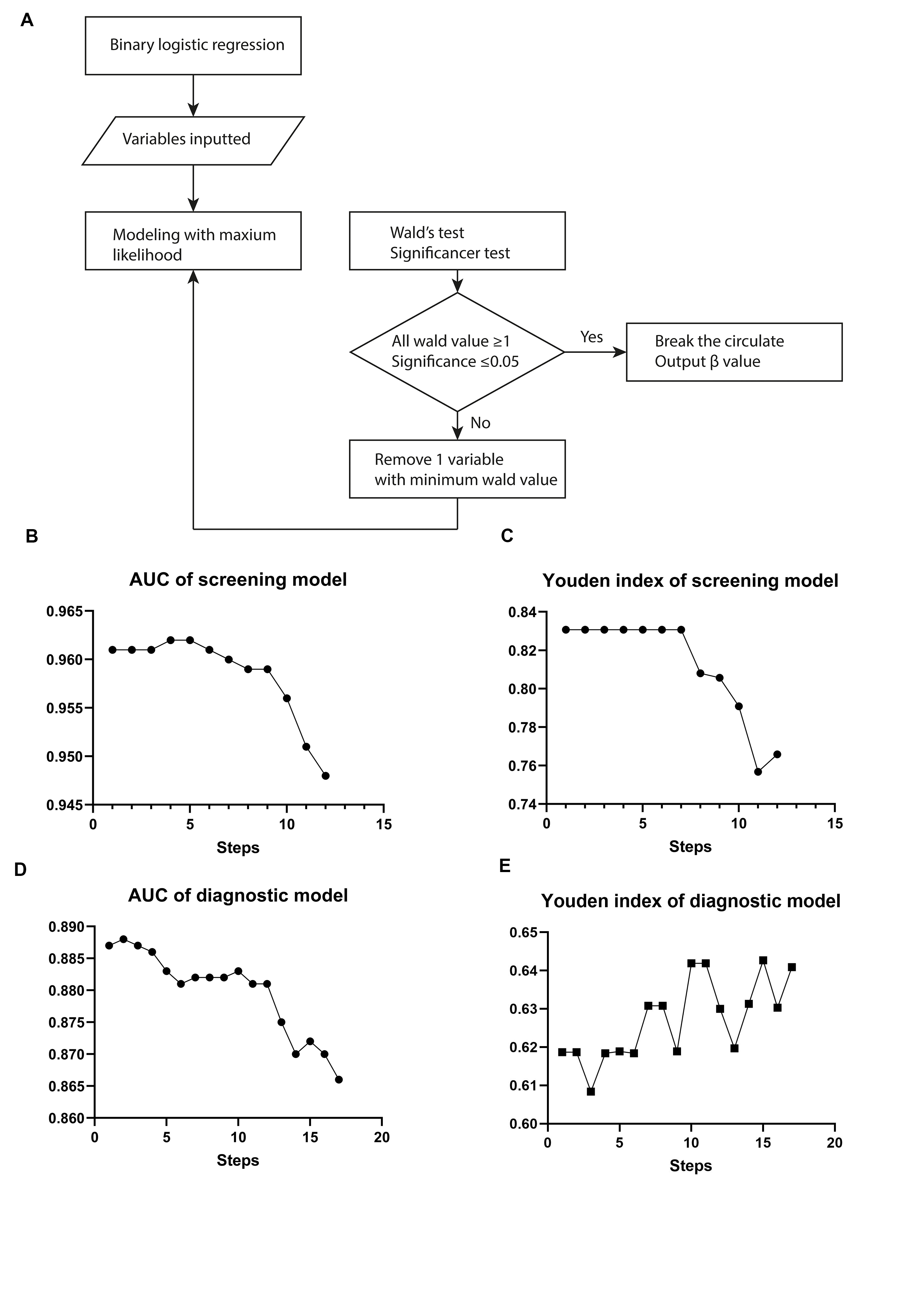


**Supplementary Figure 9: The process and the result of binary logistic regression with backward elimination methods. A.** The flow chart of backward elimination method. B. The line graph of the change in the AUC of the screening model obtained using the backward elimination method, in total of 12 steps. **C.** The line graph of the change in the Youden Index of the screening model. **D.** The line graph of the change in the AUC of the diagnostic model obtained using the backward elimination method, in total of 17 steps. **E.** The line graph of the change in the Youden Index of the diagnostic model.

**Supplementary Table 1:** Screened differentially expressed proteins and corresponding validation proteins.

| Protein  ID | Gene symbol | Protein name | Biology process | Cell component^b^ | Molecular function^c^ | KEGG  pathway^d^ | Connection node | | Candidates |
| --- | --- | --- | --- | --- | --- | --- | --- | --- | --- |
| P02647 | *APOA1* | ApoA1 | 1 | 8 | 2 | 1 | 41 | ApoA1 | |
| P02652 | *APOA2* | ApoA2 | 1 | 4 | 2 | 0 | 32 | ApoA2 | |
| P04114 | *APOB* | ApoB | 0 | 4 | 3 | 1 | 48 | ApoB | |
| P02656 | *APOC3* | ApoC3 | 0 | 3 | 2 | 0 | 22 | ApoC3 | |
| P01024 | *C3* | C3 | 3 | 5 | 6 | 4 | 51 | C3 | |
| P0C0L4 | *C4A* | C4A | 3 | 2 | 5 | 4 | 37 | C4 | |
| P0C0L5 | *C4B* | C4B | 3 | 1 | 5 | 4 | 20 | C4 | |
| P02671 | *FGA* | FGA | 7 | 8 | 1 | 2 | 45 | Fg | |
| P02675 | *FGB* | FGB | 7 | 7 | 1 | 1 | 39 | Fg | |
| P02679 | *FGG* | FGG | 7 | 8 | 1 | 1 | 47 | Fg | |
| P02751 | *FN1* | FN | 7 | 8 | 4 | 2 | 61 | FN | |
| P08519 | *LPA* | Apo(a) | 0 | 0 | 7 | 0 | 24 | Lp(a) | |
| P05155 | *SERPING1* | C1INH | 7 | 7 | 5 | 2 | 30 | C1INH | |

a): The frequency of top 10 proteins GO enrichment associated with biological process.

b): The frequency of top 10 proteins GO enrichment associated with biological cell components.

c): The frequency of top 10 proteins GO enrichment associated with molecular functions.

d): The frequency of top 10 proteins GO enrichment in KEGG pathways.

**Supplementary Table 2:** Performance of single predictor in NSCLC screening.

| Screening Index | AUC (95%*CI*) | Optimal threshold | Sensitivity (%) | Specificity (%) | Youden index |
| --- | --- | --- | --- | --- | --- |
| ApoA1 | 0.636 (0.567~0.701) | 1.36g/L | 50.91 | 73.00 | 0.24 |
| ApoA2 | 0.737 (0.672~0.759) | 23.17mg/dL | 59.09 | 82.00 | 0.41 |
| ApoB | 0.623 (0.554~0.689) | 0.89g/L | 54.55 | 65.00 | 0.20 |
| C1INH | 0.670 (0.602~0.733) | 30.90mg/dL | 55.45 | 76.00 | 0.31 |
| C3 | 0.641 (0.572~0.706) | 1.12g/L | 66.36 | 59.00 | 0.25 |
| C4 | 0.613 (0.544~0.679) | 0.24g/L | 59.09 | 58.00 | 0.17 |
| Fg | 0.689 (0.613~0.785) | 3.05g/L | 47.73 | 86.25 | 0.34 |
| FN | 0.705 (0.638~0.766) | 427.00mg/L | 55.45 | 76.00 | 0.31 |
| Lp(a) | 0.609 (0.539~0.675) | 99.40mg/L | 78.18 | 45.00 | 0.23 |
| Gly | 0.536  (0.466~0.605) | 392.96μmol/L | 30.91 | 90.91 | 0.22 |
| His | 0.744 (0.671~0.808) | 89.69μmol/L | 65.91 | 78.75 | 0.45 |
| Lys | 0.646 (0.577~0.711) | 286.36μmol/L | 81.82 | 49.00 | 0.31 |
| Tyr | 0.607 (0.538~0.674) | 75.92μmol/L | 54.55 | 70.00 | 0.25 |
| Val | 0.587 (0.517~0.654) | 262.83μm/L | 75.45 | 39.00 | 0.14 |
| Cit | 0.654 (0.586~0.719) | 29.25μmol/L | 80.91 | 44.00 | 0.25 |
| Orn | 0.655 (0.586~0.719) | 114.12μmol/L | 85.45 | 41.00 | 0.26 |
| CA | 0.602 (0.532~0.669) | 101.43nmol/L | 50.91 | 69.00 | 0.20 |
| DCA | 0.616 (0.547~0.682) | 574.43nmol/L | 85.45 | 34.00 | 0.19 |
| LCA | 0.717 (0.651~0.777) | 9.00nmol/L | 59.09 | 78.00 | 0.37 |
| UDCA | 0.656 (0.588~0.720) | 160.52nmol/L | 75.45 | 57.00 | 0.32 |
| GCDCA | 0.650 (0.582~0.715) | 2032.33nmol/L | 28.18 | 97.00 | 0.25 |
| GLCA | 0.585 (0.515~0.653) | 1.28nmol/L | 41.82 | 78.00 | 0.20 |
| TDCA | 0.687 (0.619~0.749) | 18.14nmol/L | 41.82 | 97.00 | 0.39 |
| CA19-9 | 0.594 (0.524~0.661) | 27.27U/mL | 27.27 | 92.00 | 0.19 |
| CEA | 0.724 (0.658~0.783) | 2.44ng/mL | 59.09 | 74.00 | 0.33 |
| CYFRA21-1 | 0.719 (0.653~0.778) | 2.49ng/mL | 60.00 | 77.00 | 0.38 |

**Supplementary Table 3:** Performance of single predictor in NSCLC diagnosis.

| Diagnosis Index | AUC (95%CI) | Optimal threshold | Sensitivity (%) | Specificity (%) | Youden index |
| --- | --- | --- | --- | --- | --- |
| ApoA1 | 0.717  (0.652~0.775) | 1.12g/L | 84.55 | 50.93 | 0.35 |
| ApoA2 | 0.730 (0.666~0.788) | 18.73mg/dL | 86.36 | 56.48 | 0.43 |
| ApoB | 0.639 (0.572~0.703) | 0.75g/L | 85.45 | 42.59 | 0.28 |
| ApoC3 | 0.600 (0.531~0.665) | 7.96mg/dL | 75.45 | 50.93 | 0.26 |
| C3 | 0.626 (0.558~0.691) | 1.21g/L | 53.64 | 67.59 | 0.21 |
| Fg | 0.624 (0.547~0.696) | 3.57g/L | 72.73 | 52.33 | 0.25 |
| FN | 0.669 (0.602~0.731) | 350.40mg/L | 67.27 | 58.33 | 0.26 |
| Lp(a) | 0.558 (0.489~0.625) | 308.60mg/L | 34.55 | 80.56 | 0.15 |
| Ala | 0.614 (0.546~0.679) | 446.69μmol/L | 70.91 | 50.93 | 0.22 |
| His | 0.579 (0.510~0.645) | 82.64μmol/L | 65.45 | 52.78 | 0.18 |
| Val | 0.644 (0.576~0.707) | 298.02μmol/L | 53.64 | 71.30 | 0.25 |
| Cit | 0.718 (0.653~0.777) | 28.93μmol/L | 82.73 | 57.41 | 0.40 |
| CA | 0.627 (0.559~0.692) | 173.94nmol/L | 38.18 | 85.19 | 0.23 |
| CDCA | 0.609 (0.540~0.674) | 582.60nmol/L | 50.91 | 67.59 | 0.19 |
| DCA | 0.683 (0.616~0.744) | 85.71nmol/L | 67.27 | 68.52 | 0.36 |
| UDCA | 0.620 (0.552~0.685) | 57.92nmol/L | 64.55 | 58.33 | 0.23 |
| GDCA | 0.664 (0.597~0.726) | 38.46nmol/L | 70.91 | 59.26 | 0.30 |
| GLCA | 0.576 (0.507~0.642) | 3.82nmol/L | 40.91 | 80.56 | 0.21 |
| GUDCA | 0.587 (0.519~0.653) | 84.90nmol/L | 84.55 | 43.52 | 0.28 |
| TDCA | 0.628 (0.560~0.693) | 8.57nmol/L | 79.09 | 40.74 | 0.20 |
| TLCA | 0.607 (0.539~0.672) | 0.23nmol/L | 69.09 | 52.78 | 0.22 |
| TCDCA | 0.595 (0.526~0.660) | 63.82nmol/L | 38.18 | 81.48 | 0.20 |
| CA19-9 | 0.613 (0.545~0.678) | 12.42U/mL | 50.00 | 69.44 | 0.19 |
| CEA | 0.606 (0.538~0.671) | 2.92ng/mL | 49.09 | 68.52 | 0.18 |
| CYFRA21-1 | 0.598 (0.529~0.663) | 2.85ng/mL | 53.64 | 67.59 | 0.21 |
| NSE | 0.665 (0.598~0.727) | 9.90μmol/L | 77.27 | 50.00 | 0.27 |

**Supplementary Table 4:** Screening model by stepwise binary logistic regression analysis in training samples.

| index |  | B | S.E | Wald | P | OR |  |
| --- | --- | --- | --- | --- | --- | --- | --- |
| ApoA2 |  | -0.282 | 0.115 | 6.000 | 0.014 | 0.754 (0.602～0.945) | |
| ApoB |  | 4.317 | 1.820 | 5.624 | 0.018 | 74.964 (2.115～2656.598) | |
| C3 |  | 3.948 | 1.494 | 6.982 | 0.008 | 51.812 (2.772～968.541) | |
| FN |  | -0.006 | 0.003 | 5.132 | 0.023 | 0.994 (0.989～0.999) | |
| His |  | -0.088 | 0.022 | 15.631 | 0.000 | 0.916 (0.877～0.957) | |
| Cit |  | 0.084 | 0.035 | 5.794 | 0.016 | 1.088 (1.016～1.166) | |
| Orn |  | 0.026 | 0.009 | 7.724 | 0.005 | 1.026 (1.008～1.045) | |
| CA |  | 0.001 | 0.001 | 3.048 | 0.081 | 1.001 (1.000～1.003) | |
| LCA |  | -0.071 | 0.020 | 12.135 | 0.000 | 0.932 (0.896～0.970) | |
| UDCA |  | -0.004 | 0.002 | 5.472 | 0.019 | 0.996 (0.993～0.999) | |
| GCDCA |  | 0.001 | 0.000 | 10.513 | 0.001 | 1.001 (1.000～1.002) | |
| CEA |  | 0.510 | 0.189 | 7.326 | 0.007 | 1.666 (1.151～2.411) | |
| constant |  | 2.475 | 2.988 | 0.686 | 0.408 | — | |

*B*: regression coefficient *β*; *S.E*： standard deviation; *Wald*： Wald Chi-square value; *OR*: odds ratio

**Supplementary Table 5:** Performance analysis of 3 models in screening NSCLC.

| Model | AUC (95%*CI*) | Optimal threshold | Sensitivity (%) | Specificity (%) | | Coincidence rate (%) |
| --- | --- | --- | --- | --- | --- | --- |
| Logistic Regression | 0.959 (0.917~0.983) | 0.37 | 92.05 | 88.75 | 90.48 | |
| Fisher Discriminant | 0.944 (0.898~0.973) | 0.60 | 94.32 | 85.00 | 89.88 | |
| Bayes Discriminant | 0.944 (0.897~0.973) | 0.35 | 86.36 | 91.25 | 88.69 | |

*CI*: confidence interval

**Supplementary Table 6:** Testing of 3 models in screening NSCLC.

| Model | Mistake in judgment/Test set | | | Report | | |
| --- | --- | --- | --- | --- | --- | --- |
|  | NSCLC | HC | Total | Sensitivity (%) | Specificity (%) | Coincidence rate (%) |
| Logistic Regression | 2/22 | 2/20 | 3/42 | 90.91 | 90.00 | 90.48 |
| Fisher Discriminant | 1/22 | 6/20 | 7/42 | 95.45 | 70.00 | 83.33 |
| Bayes Discriminant | 5/22 | 3/20 | 8/42 | 77.27 | 85.00 | 80.95 |

**Supplementary Table 7:** Diagnosis model by stepwise binary logistic regression analysis in training samples.

| Index | B | S.E | Wald | P | OR |  |
| --- | --- | --- | --- | --- | --- | --- |
| ApoA2 | -0.141 | 0.049 | 8.096 | 0.004 | 0.869 (0.789～0.957) | |
| Lp(a) | -0.003 | 0.001 | 6.317 | 0.012 | 0.998 (0.996～0.999) | |
| C3 | -1.949 | 0.857 | 5.170 | 0.023 | 0.142 (0.027～0.764) | |
| Fg | 0.356 | 0.206 | 2.981 | 0.084 | 1.428 (0.953～2.139) | |
| Cit | -0.058 | 0.022 | 6.929 | 0.008 | 0.944 (0.904～0.989) | |
| GDCA | -0.002 | 0.001 | 6.719 | 0.010 | 0.998 (0.996～0.985) | |
| TCDCA | 0.002 | 0.001 | 3.924 | 0.048 | 1.002 (1.000～1.004) | |
| CYFRA21-1 | -0.206 | 0.083 | 6.199 | 0.013 | 0.813 (0.691～0.957) | |
| NSE | -0.076 | 0.050 | 2.327 | 0.127 | 0.927 (0.841～1.022) | |
| constant | 8.266 | 1.761 | 22.022 | 0.000 | — | |

*B*: regression coefficient *β*; *S.E*: standard deviation; *Wald*: Wald Chi-square value; *OR*: odds ratio.

**Supplementary Table 8:** Performance analysis of 3 models in differentiating NSCLC and BPD.

| Model | AUC (95%CI) | Optimal threshold | Sensitivity (%) | Specificity (%) | | Coincidence rate (%) |
| --- | --- | --- | --- | --- | --- | --- |
| Logistic Regression | 0.871 (0.814~0.918) | 0.50 | 86.36 | 77.91 | 82.18 | |
| Fisher Discriminant | 0.859 (0.798~0.907) | 0.45 | 89.77 | 74.42 | 82.18 | |
| Bayes Discriminant | 0.855 (0.794~0.904) | 0.42 | 88.64 | 74.42 | 81.61 | |

*CI*: confidence interval.

**Supplementary Table 9:** Testing of 3 models in differentiating NSCLC and BPD.

| Model | Mistake in judgment/Test set | | | Report | | |
| --- | --- | --- | --- | --- | --- | --- |
|  | NSCLC | BPD | Total | Sensitivity (%) | Specificity (%) | Coincidence rate (%) |
| Logistic Regression | 4/22 | 3/22 | 7/44 | 81.82 | 86.36 | 84.09 |
| Fisher Discriminant | 6/22 | 2/22 | 8/44 | 72.73 | 90.91 | 81.82 |
| Bayes Discriminant | 6/22 | 3/22 | 9/44 | 72.73 | 86.36 | 79.55 |

**Supplementary Table 10:** The concentration units of these candidates.

| Candidates | Concentration units |
| --- | --- |
| Ala | μmol/L |
| Arg | μmol/L |
| Cit | μmol/L |
| Glu | μmol/L |
| Gly | μmol/L |
| His | μmol/L |
| Leu | μmol/L |
| Lys | μmol/L |
| Orn | μmol/L |
| Phe | μmol/L |
| Pro | μmol/L |
| Tyr | μmol/L |
| Val | μmol/L |
| CA | nmol/L |
| DCA | nmol/L |
| CDCA | nmol/L |
| UDCA | nmol/L |
| LCA | nmol/L |
| GCA | nmol/L |
| GLCA | nmol/L |
| GDCA | nmol/L |
| GCDCA | nmol/L |
| GUDCA | nmol/L |
| TCA | nmol/L |
| TLCA | nmol/L |
| TDCA | nmol/L |
| TCDCA | nmol/L |
| TUDCA | nmol/L |
| CA125 | U/mL |
| CA199 | U/mL |
| CEA | ng/mL |
| CYFRA211 | U/mL |
| NSE | ng/mL |
| SCC | ng/mL |
| ApoA1 | g/L |
| ApoA2 | mg/dL |
| ApoB | g/L |
| ApoC3 | mg/dL |
| C1INH | mg/dL |
| C3 | g/L |
| C4 | g/L |
| FN | mg/L |
| Lpa | mg/L |
| Fg | g/L |
